# Synergistic effects of thermally reduced graphene oxide/zinc oxide composite material on microbial infection for wound healing applications

**DOI:** 10.1038/s41598-024-73007-5

**Published:** 2024-10-03

**Authors:** A. Hassen, E. A. Moawed, Rehab Bahy, A. B. El Basaty, S. El-Sayed, Ahmed I. Ali, A. Tayel

**Affiliations:** 1 Physics Department, Faculty of Science, Fayoum University, El Fayoum, 63514 Egypt; 2 Basic Science Department, Faculty of Technology and Education, Helwan University, Saraya El Koba, El Sawah Street, Cairo, 11281 Egypt; 3 Department of Microbiology and Immunology, Faculty of Pharmacy, Fayoum University, El Fayoum, 63514 Egypt; 4 Nanotechnoloy Center, Helwan University, Helwan Al Sharqia, Cairo, 11722 Egypt; 5 Department of Applied Physics, Institute of Natural Sciences, College of Applied Science, Kyung Hee University, Suwon, 446-701 Republic of Korea

**Keywords:** TRGO/ZnO nanocomposites, Hydrothermal synthesis, Characterizations, Optical properties, Antibacterial activity, Pathogenic microorganism, Biological techniques, Materials science

## Abstract

Infections originating from pathogenic microorganisms can significantly impede the natural wound-healing process. To address this obstacle, innovative bio-active nanomaterials have been developed to enhance antibacterial capabilities. This study focuses on the preparation of nanocomposites from thermally reduced graphene oxide and zinc oxide (TRGO/ZnO). The hydrothermal method was employed to synthesize these nanocomposites, and their physicochemical properties were comprehensively characterized using X-ray diffraction analysis **(**XRD**)**, High-resolution transmission electron microscopy (HR-TEM), Fourier-transform infrared (FT-IR), Raman spectroscopy, UV-vis, and field-emission scanning electron microscopy (FE-SEM) techniques. Subsequently, the potential of TRGO/ZnO nanocomposites as bio-active materials against wound infection-causing bacteria, including *Staphylococcus aureus*, *Pseudomonas aeruginosa*, and *Escherichia coli*, was evaluated. Furthermore, the investigated samples show disrupted bacterial biofilm formation. A reactive oxygen species (ROS) assay was conducted to investigate the mechanism of nanocomposite inhibition against bacteria and for further in-vivo determination of antimicrobial activity. The MTT assay was performed to ensure the safety and biocompatibility of nanocomposite. The results suggest that TRGO/ZnO nanocomposites have the potential to serve as effective bio-active nanomaterials for combating pathogenic microorganisms present in wounds.

## Introduction

The skin, the body’s largest and outermost organ, serves as a protective barrier for internal organs, muscles, and bones against the external environment^1^. It plays a vital role in preventing dehydration, sensory perception, detection, and temperature regulation. Skin can be damaged by various means, including chemical, physical, and microbial factors, resulting in wounds^2^. The primary concern is wound infections caused by microorganisms, as wounds provide an ideal environment for microbial growth and proliferation, ultimately hindering wound healing^3,4^. Gram-positive bacteria like *Staphylococcus aureus* are typically present during the initial stages of wound development, while gram-negative bacteria such as *Escherichia coli* found in later stages^5^. The inability of the immune system to eliminate these microorganisms and the development of microbial resistance pose significant challenges. Advanced nanomaterials are emerging as a promising approach in the field of medical applications^6,7^.

Nanomaterials have emerged as powerful antimicrobial agents, leveraging their unique nanostructures to combat bacterial and microbial growth effectively^8^. The intrinsic properties of these nanoscale structures, such as high surface area-to-volume ratios and enhanced reactivity, enable them to interact with microorganisms at a fundamental level. By exploiting these properties, nanomaterials can disrupt the cell membranes of bacteria and interfere with their metabolic processes, ultimately inhibiting bacterial growth and proliferation^9^. Additionally, nanomaterials can be engineered to release antimicrobial agents in a controlled manner, ensuring a sustained and targeted approach to combating infections. This innovative use of nanotechnology holds great promise in various applications, from medical devices to environmental sanitation, as it offers a potent and precise means of reducing bacterial and microbial impact, thereby enhancing overall public health and safety^10,11^.

Bio-nanomaterials make use of a wide variety of inorganic nanomaterials, including metal and metal oxide nanoparticles, carbon-based nanoparticles, and ceramic nanoparticles^12,13^. These materials collectively confer favorable bioactive, mechanical, and bactericidal attributes, contributing synergistically to heightened therapeutic efficacy in wound treatment^3,14,15^. Zinc oxide (ZnO), among metal oxides, stands out for its potential in wound healing applications, owing to its antimicrobial properties facilitated by the generation of reactive oxygen species (ROS) upon exposure to ultraviolet (UV) light^16,17^. This unique feature assists in preventing infections in wounds. ZnO exhibits biocompatibility, characterized by low toxicity and anti-inflammatory properties, making it suitable for medical use. Furthermore, ZnO nanoparticles play a supportive role in cell proliferation and migration, promoting tissue regeneration^18,19^. Despite these advantages, challenges exist, including potential cytotoxicity at higher concentrations and concerns regarding the long-term impact of nanoparticle accumulation in organs or tissues. While ZnO holds promise in wound healing, careful consideration of these challenges is crucial for its safe and effective applications^20,21^.

Carbon base nanomaterials, especially graphene, have diverse unique physical and fascinating chemical properties such as exceptional mechanical strength, high electrical conductivity, excellent thermal conductivity, large surface area per unit mass, chemical stability, extremely lightweight, flexible, and biocompatibility^22,23^. Besides those properties, graphene has an antibacterial action due to its hydrophobic nature and the generation of reactive oxygen species (ROS)^24,25^. When interacting with bacteria, nanomaterial substantially catalyzes ROS-independent oxidation, demonstrating good antibacterial activity. Graphene-based materials display impressive antibacterial properties^26,27^, thanks to three key mechanisms. Firstly, they provoke oxidative stress (OS), which targets and reduces bacteria. Secondly, the sharp edges of the material encourage cell induction and delay bacterial growth. Finally, they efficiently trap bacteria, restricting their metabolism rate and impeding their physical mobility. These multifaceted mechanisms truly emphasize the immense potential of graphene in effectively combating bacterial infections^28^.

Recent studies have highlighted the remarkable antibacterial properties of carbon-based nanomaterials, such as graphene oxide (GO) nanoparticles and carbon nanotubes (CNTs)^29–31^. Combining these carbon-based materials with silver (Ag) has demonstrated highly effective inhibitory effects against critical bacteria, including *Burkholderiacepacia*, *Klebsiella pneumonia*, and *S. aureus*^32,33^. Moreover, these composite nanomaterials have shown promise in inhibiting the growth of bio-defense bacteria like *Yersinia pestis*^34–36^.

The smaller size and higher density of functional groups in graphene nanoparticles enhance their interaction with bacterial cells, leading to cell deposition. This interaction can alter DNA and rupture cell membranes, inducing membrane stress and ultimately causing cell death. Notably, graphene nanocomposites exhibit a significant time and concentration-dependent ability to oxidize glutathione (GSH), a crucial element for eliminating various reactive species. This oxidative stress (OS) induced by graphene nanocomposites is not solely dependent on superoxide anions, distinguishing them from other materials. Moreover, conductive reduced graphene oxide (TRGO) may mediate more potent oxidative stress through direct interaction with cells when compared to insulating GO^37–39^. This strategic combination of carbon-based materials enhances the bioactivity, antimicrobial, and antibacterial activities of the resulting nanocomposite, making it a promising candidate for various applications in biomedical and antibacterial research.

The composition of reduced graphene (RG) with zinc oxide (ZnO) provides a substantial advantage for applications requiring antibacterial and antimicrobial materials. Rajeswari et al.^40^ led a novel approach utilizing a hydrothermal technique that has been employed to cultivate ZnO nano-rods in conjunction with RG, resulting in a notable enhancement of antibacterial effects. However, despite these encouraging findings, a significant challenge arises from the morphology of the ZnO rods, which tend to exhibit a relatively large and randomly distributed structure. This variance in morphology can directly impact the composite’s antibacterial performance.

Furthermore, another study has explored the antibacterial activities of similar composite variations^41^. It was observed that the results depend on the specific preparation techniques. These varieties underscore the need for further investigation and optimization of the composite’s antibacterial properties. By delving deeper into the underlying mechanisms and refining the synthesis methods, researchers can strive to standardize and enhance the antibacterial efficacy of the GO/ZnO composite. Such efforts are essential to unlock the full potential of this composite for various applications, including biomedical devices, water purification systems, and antimicrobial coatings.

The primary goal of this study is to harness the synergistic antibacterial properties of thermally reduced graphene oxide (TRGO) and ZnO while addressing potential toxicity concerns associated with higher ZnO quantities. In our approach, we utilize the hydrothermal technique to decorate TRGO with ZnO material, aiming to optimize antibacterial performance while minimizing adverse effects. This strategy not only capitalizes on the intrinsic antibacterial efficacy of TRGO, derived from carbon atoms but also addresses the potential toxicity linked to elevated ZnO quantities. Our thorough analysis encompasses the structure, morphology, and FTIR results, confirming the successful synthesis. Additionally, we investigate optical properties and discuss the implications. The biological analysis further supports our findings, revealing that the TRGO/ZnO composite exhibits superior antibacterial properties compared to using bare ZnO or graphene alone. This comprehensive approach positions the composite as a promising advancement for applications requiring robust antibacterial features, particularly in wound healing procedures where minimizing nanomaterial amounts is crucial to preventing potential toxicity.

## Experimental work

### Materials

The chemicals used in this study were bought from various suppliers. Zinc powder (0.65 μm), graphite powder (GP), and methylene blue (MB) were obtained from Sigma Aldrich, while potassium permanganate, barium chloride, hydrogen peroxide, sulfuric acid, hydrochloric acid, tetrahydrofuran, acetone, along with oxygen and hydrogen gases, were acquired from Ducksan Chemicals. All chemicals were used as received, without further purification. Deionized (DI) water was employed throughout the investigation. The antibacterial activity of the materials was evaluated against two gram-negative bacterial strains, *Pseudomonas aeruginosa* and *Escherichia coli*, and one gram-positive strain, *Staphylococcus aureus*, for both individual and mixed nanomaterials.

**Fig. 1:**
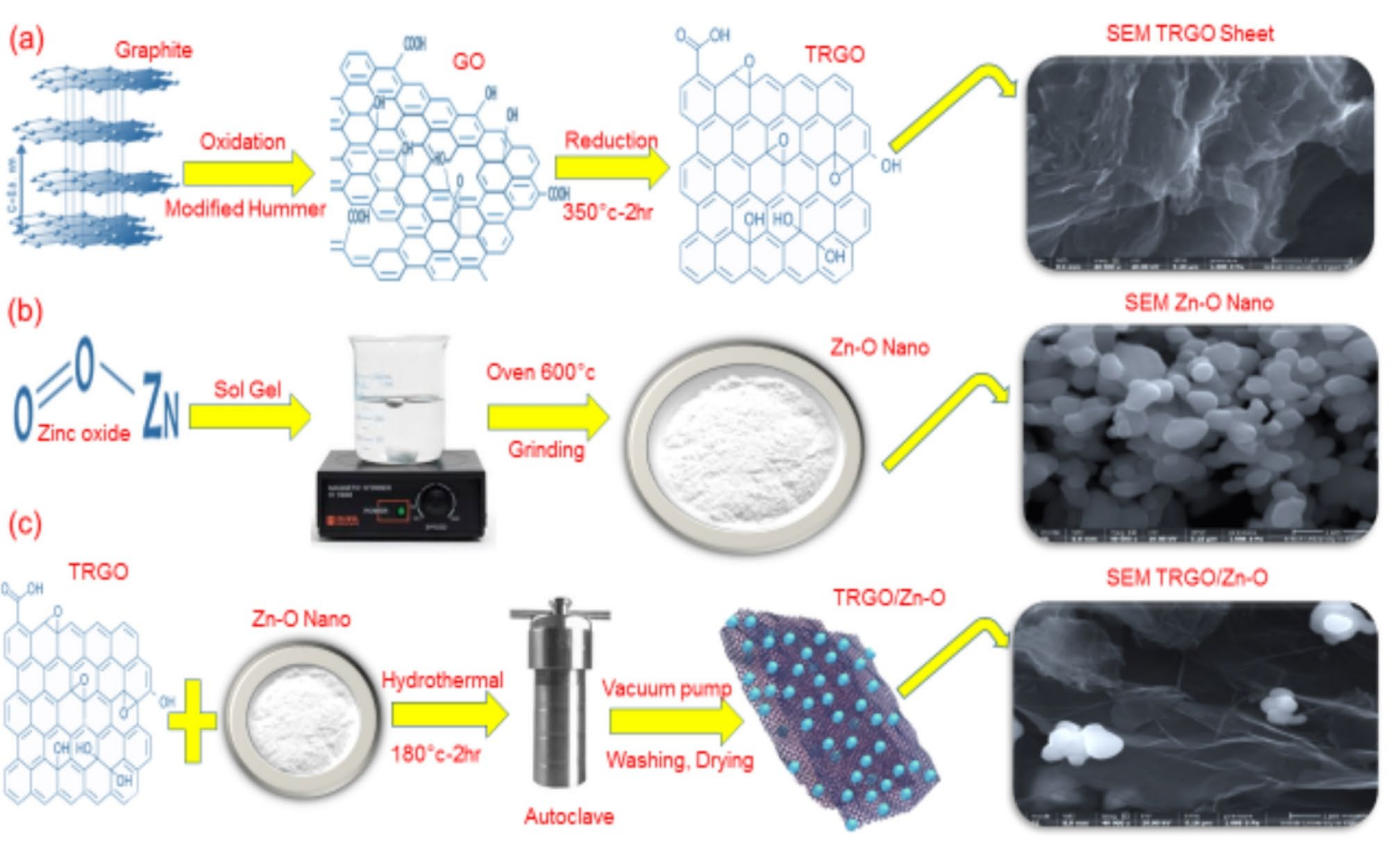
**A** schematic preparation route, (**a**) thermal reduced graphene oxide (TRGO), (**b**) zinc oxide nanoparticles (ZnO), (**c**) TRGO/ZnO nanocomposite.

### Samples synthesis

#### Synthesis of Graphene oxide (GO)

Graphene oxide (GO) was produced by modifying Hummers’ technique^42^. Initially, 2.0 g of graphite flakes and 2.0 g of NaNO_3_ were added to 46 ml of concentrated H_2_SO_4_ and stirred for 2 h. The temperature was maintained at 0 °C by placing the mixture in an ice bath. Subsequently, 3.0 g of KMnO_4_ was added gradually to the stirring solution while keeping the temperature below 20 °C. As a result, the solution’s color changed from black to brown. Further, the stirring was continued by raising the temperature to 35 °C for 30 min, and then 92 ml of distilled water was slowly added to it where heat was liberated. Then 150 ml of distilled water in one shot. After a minute of stirring, 20 ml of H_2_O_2_ is added to the above solution. The solution’s color shifts from brown to greenish-yellow, signaling the completion of the reaction. The mixture is then subjected to ultrasonication and washed multiple times with distilled water, 5% HCl, and acetone. Afterward, the sample is dried in a hot air oven at 60 °C for 2 h before being collected. The summary of the sample preparation via modifying Hummer’s method is depicted schematically in Fig. 1(a).

### Synthesis of Zinc Oxide (ZnO)

ZnO was synthesized by using the sol-gel method. A solution of 20 g of ZnO dissolved with 400 ml methanol was prepared. Then, the solution was stirred in the hot plate of a magnetic stirrer at a temperature of 60 to 70 °C for about an hour until the evaporation of methanol, leaving a white precipitate. The white precipitate was grinding well for two hours and put on a plate into the oven at a temperature (500–700 degrees) for 2 h. Finally, it is grinding well again. Figure 1(b) provides a succinct overview of the ZnO sol-gel preparation method^43^.

###  Synthesis of TRGO/ZnO

TRGO/ZnO nanocomposites with 1:1 wt% ratio were synthesized via the hydrothermal method^44^. ZnO and TRGO nanoparticles, along with ethanol, were used as starting materials. Initially, 0.114 mg of TRGO powder was dispersed in 50 mL of ethanol, while 0.112 mg of ZnO was dispersed in 50 mL of ethanol. The two solutions were then thoroughly mixed using an ultrasonic bath for 1 h at room temperature. The mixture was transferred to a 75 mL Teflon-lined autoclave and treated at 180 °C for 2 h. The resulting products were centrifuged using a vacuum pump, washed with ethanol and distilled water, and then dried at 60 °C for 12 h (see Fig. 1(c)).

### Biological Activity

#### Antimicrobial activity

Plate count assay was used to evaluate the antimicrobial activity of the three nanoparticles TRGO, ZnO, and TRGO/ZnO. Briefly, *E. coli* ATCC10536, *Ps. aeruginosa* ATCC9027 and *S. aureus* ATCC9144 were cultured in Luria-Bertani (LB) medium at 37 °C. Using a double-beam spectrophotometer, the sub-culture was diluted to reach 0.2 OD600 which contained 10^6^ colony-forming units (CFU/mL). Approximately 1 × 10^7^ CFU/mL of overnight bacterial cultures was added to 20 mL of Mueller Hinton broth (MHB) in 50 mL flasks for the antimicrobial activity assay. 0.2 g of each of the three nanoparticles made of TRGO, ZnO, and TRGO/ZnO were added to each bacterial flask, and cultures were serially diluted to obtain final concentrations of 0.01, 0.005, 0.0025, 0.00125, and 0.000625 g/ml. Bacterial cells were cultured for 24 h at 37 °C, cultures without nanoparticle treatment were used as control, each concentration was placed on three LB agar plates, and incubated at 37 °C overnight, and cell counts were performed. Each value is the meaning of three technical replicates. One-way analysis of variance was used to examine statistical significance (ANOVA)^45,46^.

### Biofilm reduction with TRGO, ZnO, and TRGO/ZnO nanocomposite

The biofilm formation was inhibited using a 96-well Polystyrene microliter with a flat bottom plate loaded with 100 µL of Nutrient Broth (NB) medium containing 0.01% of each dispersed TRGO, ZnO, and TRGO/ZnO nanocomposite. Separately, in each well of the plate, 2.5 µL of the respective bacterial suspension (1 × 10^7^ CFU) was added. As a negative control, each bacterial strain was suspended in 100 µL without properly the addition of nanomaterials. The positive control, 100 µL of NB medium containing 0.01% of each of the nanomaterials was adjusted. The plate was incubated in a shaking incubator at 30 °C for 24 h. Following the period of incubation, the contents of each plate well were collected out, twice cleaned by phosphate-buffered saline (PBS), and then stained with 0.1% crystal violet for 30 min (150 µL). The plate was then rinsed with distilled water to remove any remaining discolorations from each well. The stain was dissolved in 125 µL of 33% (v/v) acetic acid for 15 min. The optical density (OD) was measured at 540 nm with a microplate spectrophotometer. Eradication % was estimated using the following equation^45^.

Percentage (%) inhibition = OD Negative control- OD Sample X 100/OD Negative control^47^.

### Reactive oxygen species (ROS) assay

The ROS generation was assessed using the oxidant-sensitive dye DCFH-DA (2′,7′-Dichlorodihydrofluorescein diacetate). Cultures of bacteria were added to TRGO/ZnO nanocomposite and mixed properly; they were incubated for three hours. Each culture has been centrifuged in increments of 1–2 mL for 5 min at 5000 rpm. The supernatant was removed, and the pellet was suspended in PBS. A 30 µg/ml of DCFH-DA was added and incubated for 45 min at 37 °C. The positive control was treated with H_2_O_2_ for 30 min before adding DCFH-DA. Other cells that were not treated acted as a negative control. Fluorescence intensity was determined using a microplate reader (Readwell Touch, India ), the excitation wavelength is 500 nm, and the emission wavelength is 525 nm. The degree of intracellular ROS is correlated with the fluorescence intensity^48^.

### Evaluation of cytotoxicity and cell viability of TRGO/ZnO nanocomposite (MTT assay)

The MTT assay was used to assess the cytotoxicity of TRGO/ZnO nanocomposite in a primary culture of skin cells (Normal Human Epidermal Keratinocytes (NHEK)) (C-12003, Sigma Aldrich, USA). Briefly, A 96-well tissue culture plate was filled with 1 × 10^5^ cells / mL (approximate 100 µL/well) and cultivated for 24 h at 37 °C to create a complete monolayer sheet, following the formation of a confluent sheet of cells, the growth material was removed from the 96-well microtiter plates, and the cell monolayer was twice washed using wash media, 2% serum (maintenance medium) was added to RPMI medium to create two-fold dilutions of the tested sample ranging from 1000 to 31.25 µg/ml. Each dilution was examined in 0.1 mL increments in another three wells acting as controls and receiving only the maintenance media. The plate was incubated for 24 h at 37 °C and examined for physical indicators of toxicity, as shrinkage, rounding, granulation, or partial or whole loss of the monolayer, was examined within the cells. A total of 5 mg/ml MTT solution was made in PBS (BIO BASIC, CANADA INC). To every well, 20 uL of MTT solution was added, and then was shaken at 150 rpm for five minutes to fully incorporate the MTT. MTT was allowed to be metabolized for four hours by incubating at 37 °C with 5% CO_2_ to create crystals of formazan. Formazan crystals were then dissolved with 200 µL of DMSO added to each well. Optical density was read at 560 nm and subtracted background at 620 nm^49,50^.

### Samples characterizations

X-ray diffraction (XRD) patterns of the prepared samples were obtained using a PANalytical Empyrean 3rd generation XRD system with Cu-Kα radiation (λ = 1.540 Å) at 20 kV. A detector step size of 0.02° s⁻¹ was used for the scans, which covered an angular range of 2θ from 10° to 80°. The surface morphology of the samples was analyzed using field emission scanning electron microscopy (FE-SEM) (Thermo Scientific, Quattro SEM). Additionally, high-resolution transmission electron microscopy (HR-TEM) was performed with a JEOL-JEM 2100 instrument for further characterization. Optical spectra were recorded using an Agilent Cary 5000 UV-Vis spectrophotometer, measuring reflectance over the wavelength range of 300 nm to 800 nm. Optical parameters were calculated using empirical equations^51^. Finally, Raman spectroscopy was performed using a Confocal Raman Microscope (WiTec,300R alpha, made in Germany), Raman spectrograph with laser power of 1mW and 532 nm wavelength.

### Results and discussion

#### X-ray diffraction (XRD)

**Fig. 2:**
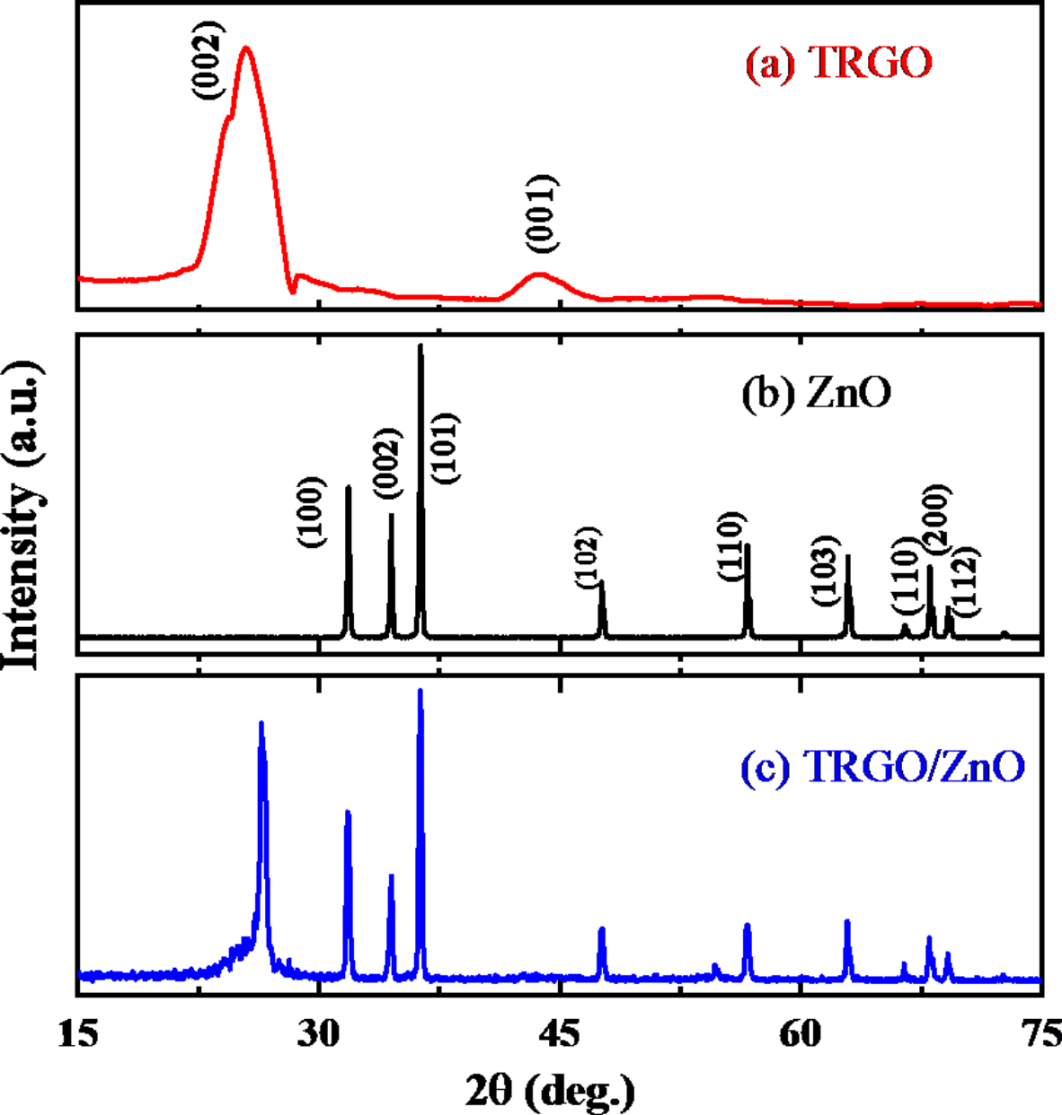
X-ray diffraction patterns of (**a**) thermally reduced graphene oxide (TRGO), (**b**) pure zinc oxide (ZnO), and (**c**) the TRGO/ZnO nanocomposite.

Figure 2 (a-c) illustrates the XRD patterns of the as-prepared samples. In the XRD smooth pattern of TRGO shown in Fig. 2 (a), two distinctive peaks are visible, with the first peak representing the (002) plane observed at 2θ = 25.4°, confirming the complete reduction of graphene oxide (GO) through thermal treatment^52^. The peak’s presence suggests the successful and complete reduction of GO, a crucial step in obtaining TRGO. The second peak, located at approximately 2θ = 43.6°, corresponds to the (100) plane of the hexagonal carbon structure^53^. Figure 2 (b) illustrates the XRD pattern of the ZnO nanoparticles^54,55^. The diffraction peaks matched the established ZnO standard, JCPDS card no. 36-1451, confirming the presence of a hexagonal-phase wurtzite crystal structure in the synthesized ZnO. This structural identification is a crucial aspect of our analysis, as it defines the fundamental crystalline nature of the prepared ZnO^56–58^. Besides, the XRD pattern of ZnO nanoparticles deposited onto the TRGO nanosheet using the hydrothermal technique is depicted in Fig. 2 (c). Notably, the characteristic diffraction peaks of ZnO are evident in this pattern, although with reduced intensity compared to Fig. 2 (b). This decrease in intensity can be attributed to the relatively small quantity of ZnO particles incorporated in the composite material. The XRD analysis reveals an increase in the (002) peak associated with TRGO, which suggests a significant interaction between the ZnO nanoparticles and the TRGO nanosheet. This interaction is indicative of the successful decoration of the TRGO nanosheet with ZnO, a crucial observation for understanding the structural changes brought about by this combination^59–61^.

### FE-SEM characterization

**Fig. 3:**
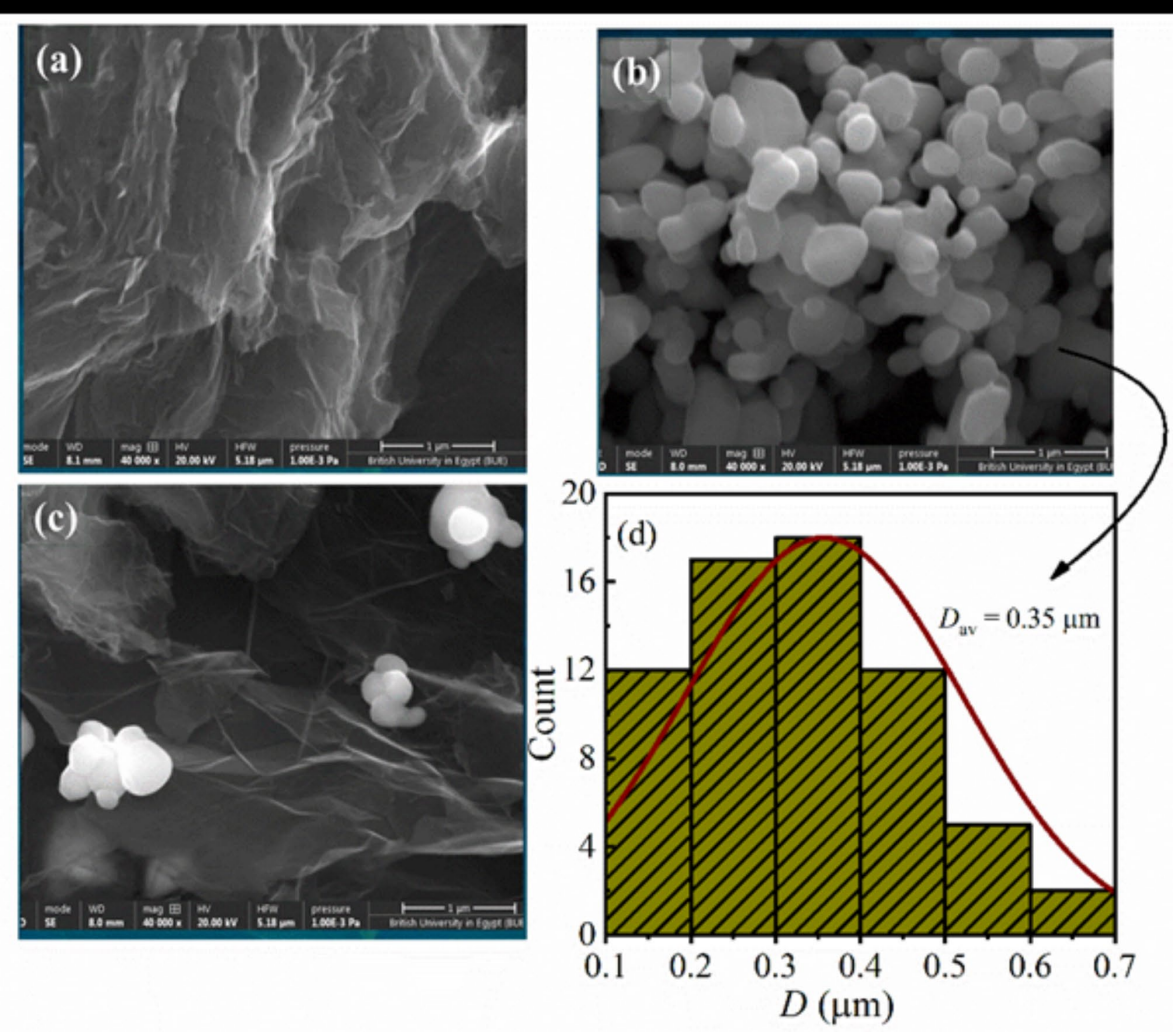
**(a–d)**: FE-SEM images of (**a**) thermally reduced graphene oxide (TRGO), (**b**) pure zinc oxide (ZnO), (**c**) the TRGO/ZnO nanocomposite and (**d**) the particle size distribution histogram of ZnO particles.

Understanding the morphology of the prepared samples is crucial for comprehending the material’s structure and behavior. Figure 3 presents the FE-SEM images of the prepared samples, with Fig. 3 (a) depicting the FE-SEM image of the TRGO material. The thermally reduced graphene oxide exhibits a unique structural arrangement, featuring interconnected, crumpled graphene sheets. These sheets form a highly porous and three-dimensional network, characterized by irregularly contoured, wrinkled edges and occasional agglomerated regions. Notably, individual graphene sheets often display a crumpled or wrinkled appearance, a direct outcome of the thermal reduction process^61,62^. In contrast, Fig. 3(b) provides the morphology of the ZnO material. The ZnO particles possess a predominantly near-spherical shape, but what’s particularly noteworthy is the distinct size distribution observed within the sample. These particles vary in size, with an average particle size of approximately 0.35 μm (see Fig. 3 (d)). In addition, Fig. 3 (c), depicts the adorning of ZnO particles on the graphene nanosheet. A small cluster of ZnO particles is noticeable, adhering to a crumpled graphene sheet^63,64^.

### HR-TEM characterization

**Fig. 4:**
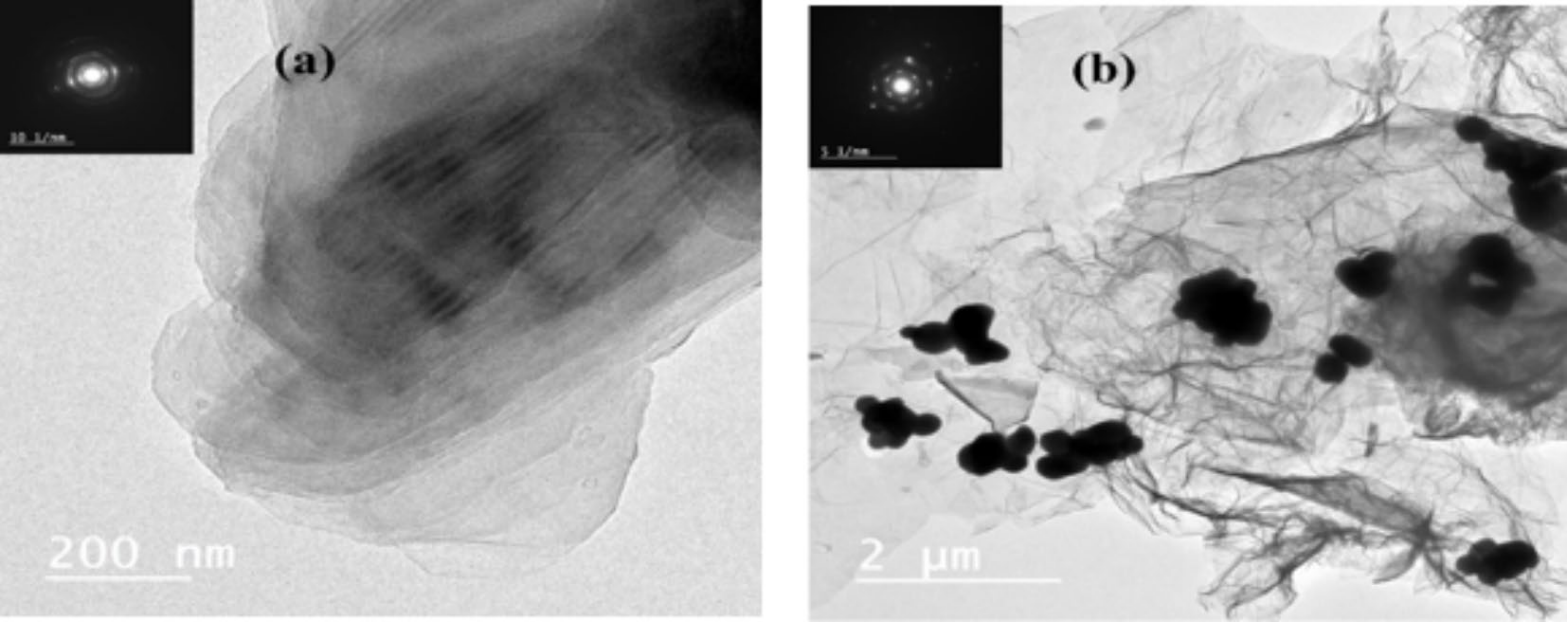
**(a-b)**: HR-TEM images of (**a**) thermally reduced graphene oxide (TRGO) and (**b**) the TRGO/ZnO nanocomposite. The insets show the corresponding SAED patterns for (**a**) TRGO and (**b**) TRGO/ZnO.

The HR-TEM image in Fig. 4(a) reveals the microstructure of thermally reduced graphene oxide (TRGO), displaying a large graphene sheet with characteristic wrinkles, a result of the thermal reduction process. These wrinkles are indicative of the structural changes that occur when oxygen-containing functional groups are removed during heat treatment, leading to a more folded and crumpled morphology. Besides, in Fig. 4(b), the incorporation of ZnO nanoparticles onto the TRGO sheet is observed. The ZnO particles are small clusters distributed across the graphene sheet, demonstrating effective interaction and attachment between the ZnO nanoparticles and the TRGO surface. This distribution is crucial for enhancing the composite’s properties, as it maximizes the surface area and ensures the consistent existence of ZnO on the graphene matrix.

### FT-IR characterization

**Fig. 5:**
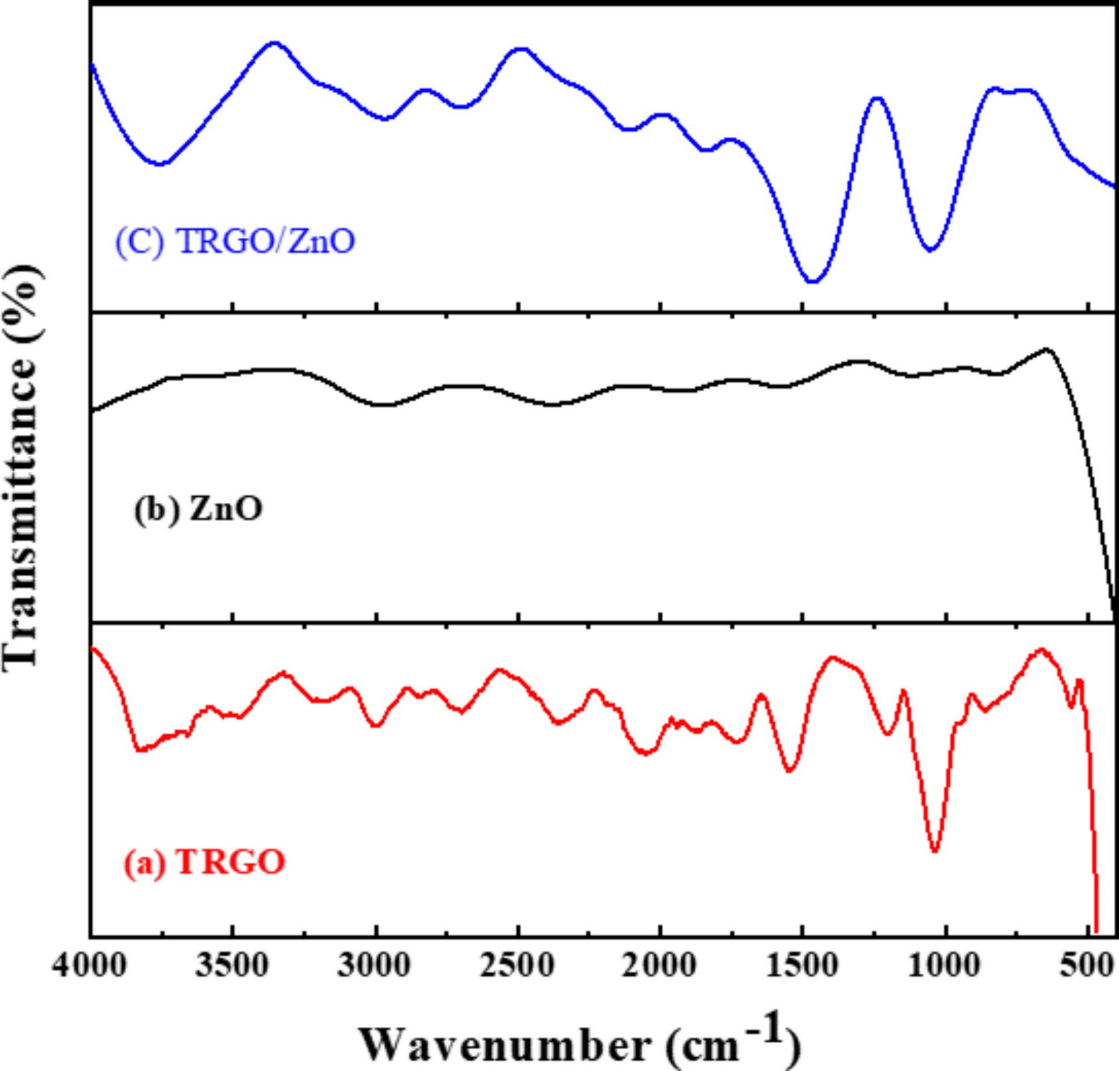
**(a-c)**: FT-IR for the (**a**) thermally reduced graphene oxide (TRGO), (**b**) pure zinc oxide (ZnO), and (**c**) the TRGO/ZnO nanocomposite.

Figure 5 represents the FT-IR spectra for three distinct materials: thermal reduced graphene oxide (TRGO), pure (ZnO), and the resulting TRGO/ZnO nanocomposite. In Fig. 5 (a), the FT-IR spectrum of TRGO is presented, offering insights into the sample’s functional groups. Graphene oxides are known to contain a variety of oxygen functional groups, such as hydroxyl, carbonyl, and alkene functional groups. The FT-IR spectrum shows a noticeable drop in several oxygen functional groups upon reduction of graphene oxide to TRGO. More specifically, the distinctive bond forms in graphene oxide are indicated by the existence of C-O, O-H, C-O-C, C = C, and C-H bonds. The TRGO FT-IR spectrum showed peaks that match oxygen bond stretching vibrations, suggesting that the reduction process may not have been completed. Peaks are evident at 1000 cm^-1^,1200 cm^-1^,1500 cm^-1^,2000 cm^-1^, 2750 cm^-1^, 3400 cm^-1^, and 3450 cm^-1^, suggesting the presence of residual hydroxyl groups (O-H), (C–O), and (C-H) in the layers of TRGO. This persistence of certain functional groups can be attributed to an incomplete reaction with these groups during the reduction process, contributing to the imperfect removal of specific oxygen bonds^65,66^.

Figure 5 (b) displays the bonding between Zn and O in the 400 to 4000 Cm^− 1^ range. As can be seen from the curve of the spectrum, there are 3 peaks at 550, 505, and 3000 Cm^–1^. The first peak at 550 Cm^–1^ is related to the ZnO bonds. The second peak around 505 Cm^-1^ is associated with oxygen deficiency and/or oxygen vacancy (V_O_) defect complex in ZnO. The third peak around 3000 attributed to the C = O bond vibrations. The experimental results are in good agreement with previous research^67–71^. Figure 5 (c) shows some of the bonds of both spectrums of the ZnO and TRGO vibrations. The bonds such as C-O, C-C, O-H, and ZnO appeared in the FT-IR spectra. However, the FE-SEM image shows the ZnO nanoparticle over the graphene well, and the sheets of graphene cross-linked to the nanoparticles of ZnO.

### Raman Spectroscopy

**Fig. 6:**
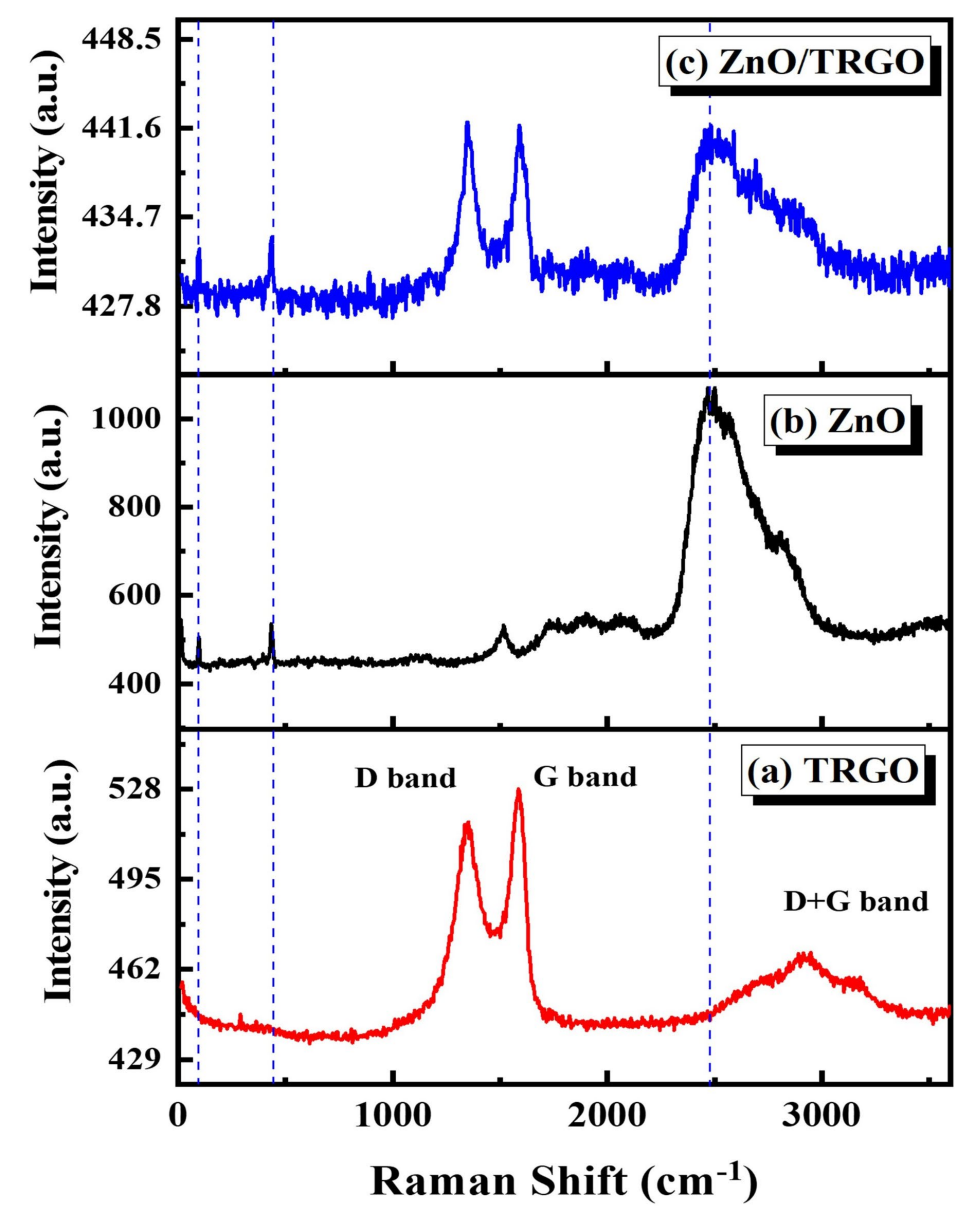
**(a-c)**: Raman Spectra for the (**a**) thermally reduced graphene oxide (TRGO), (**b**) pure zinc oxide (ZnO), and (**c**) the TRGO/ZnO nanocomposite.

Figure 6(a-c) shows the Raman spectra of the samples under investigation. In Fig. 6(a), the Raman spectrum of TRGO is presented, featuring prominent D-band and G-band peaks at 1343 and 1594 cm^− 1^, respectively. The D-band is associated with the presence of defects in the graphene lattice, arising from the breathing modes of sp^2^ carbon atoms in rings. This band is commonly used to evaluate the level of defects or functionalization in graphene. In contrast, the G-band corresponds to the in-plane vibrations of sp^2^ carbon atoms, a characteristic peak of graphitic materials, which signifies the presence of graphitic domains in the reduced graphene oxide. Additionally, a peak at 2924 cm^-1^ is observed, corresponding to the combination of D and G modes. The presence of this band further emphasizes the existence of defects and disorder within the graphene structure. A crucial parameter for evaluating the degree of reduction and disorder in graphene is the intensity ratio of the D band to the G band (I_D_ /I_G_). In the Raman spectrum of TRGO, as shown in Fig. 6(a). This ratio is determined to be 0.98, indicating defects /disorders in the graphene lattice that were introduced during the reduction process.

Figure 6(b) presents the Raman spectrum of ZnO material. Two intrinsic vibrational modes of ZnO are observed at 100 cm^− 1^ and 437 cm^− 1^, corresponding to the E_2_ and E_2_^H^ modes, respectively. The mode is associated with the low-frequency branch of the E_2_ mode, involving vibrations of the zinc sublattice. In contrast, the E_2_^H^ mode is a non-polar optical phonon mode and is the most intense Raman peak for ZnO, attributed to the vibrations of oxygen atoms. The large peak observed at 1478 cm^−1^ is atypical for the intrinsic vibrational modes of ZnO, such as those associated with its wurtzite crystal structure. This peak is likely related to the presence of defects, impurities, or surface effects, particularly in nanoparticle forms of ZnO. The presence of oxygen vacancies, zinc interstitials, or other defects can introduce additional vibrational modes that deviate from the intrinsic phonon modes of ZnO, often appearing at higher frequencies. Additionally, this peak could originate from surface phonon modes, which may arise due to the large surface-to-volume ratio in nanoscale materials. These surface-related modes typically appear at different frequencies compared to bulk material and can be shifted to higher wavenumbers.

The Raman spectrum of the TRGO/ZnO composite is displayed in Fig. 6(c). The spectrum clearly shows the convolution of characteristic peaks from both TRGO and ZnO, reflecting the successful integration of the two materials. Notably, the I/I_*G*_ ratio is calculated to be 1, indicating that the defects on the TRGO surface are significantly reduced, likely due to the decoration of ZnO particles on its surface. This reduction in defects suggests that the interaction between ZnO and TRGO has a stabilizing effect on the graphene structure.

### Optical properties

**Fig. 7:**
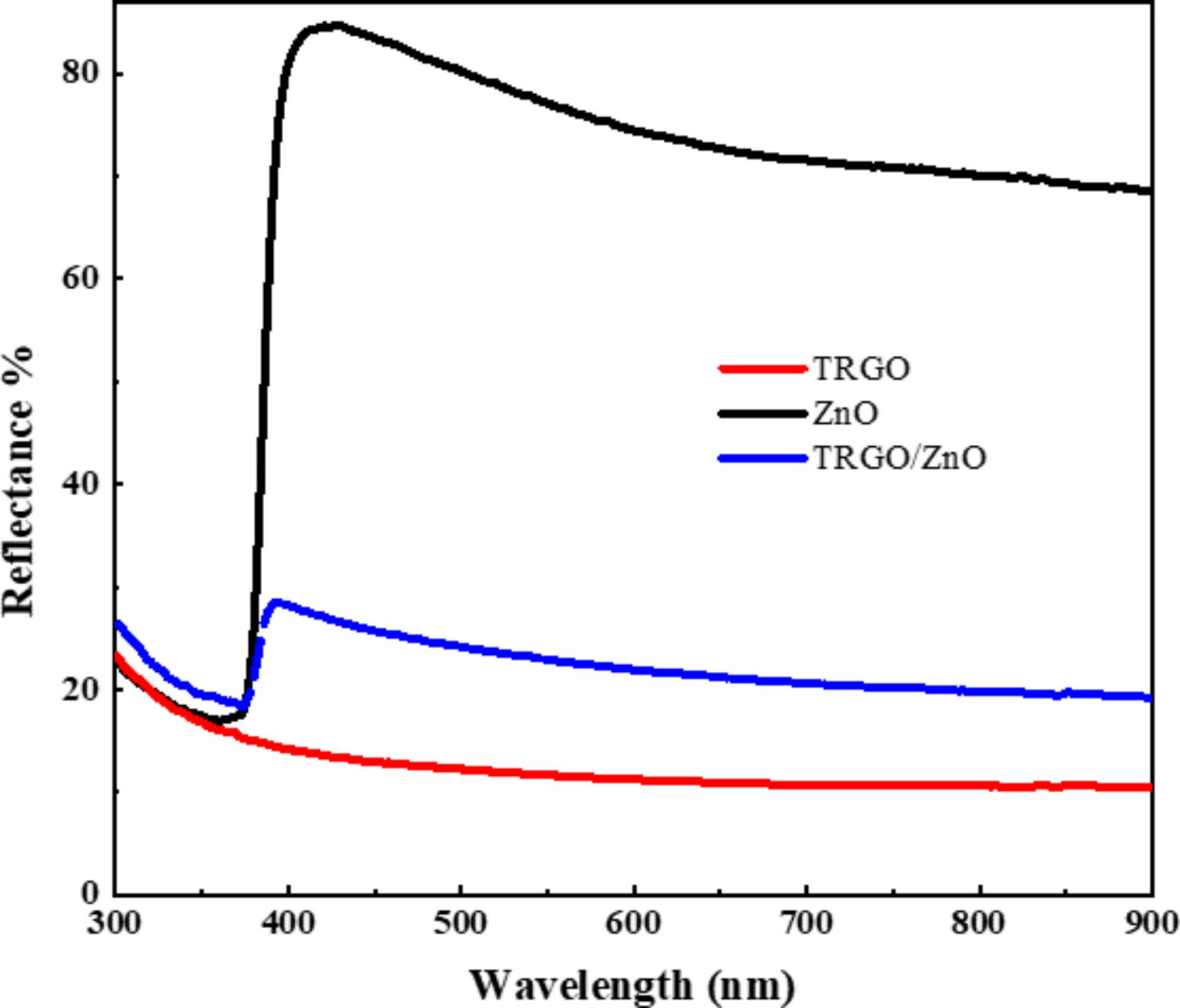
Reflectance as a function of the wavelength for TRGO, ZnO, and TRGO/ZnO samples.

The optical properties of the as-prepared samples were assessed via diffuse reflectance UV-visible spectra spectroscopy at room temperature. Utilizing established equations^40^, optical parameters were derived from the gathered data. Figure 7 illustrates the wavelength-dependent reflections of ZnO, TRGO, and TRGO/ZnO samples. Analysis of the experimental optical reflection data reveals low reflections at wavelengths below 380 nm, with a notable increase in reflections at higher wavelengths. Specifically, the reflection of ZnO approaches approximately 90%, whereas, for TRGO/ZnO samples, it diminishes to approximately 25–30% within the visible spectrum range. This reduction in reflection can be attributed to the mitigating effect of TRGO on the reflective properties of ZnO, attributable to the inherent characteristics of graphene. Notably, in the region (λ > 380 nm), reflections exhibit independence from photon energy, demonstrating consistent reflection across all samples irrespective of wavelength, as corroborated by previous studies^72–74^.

**Fig. 8:**
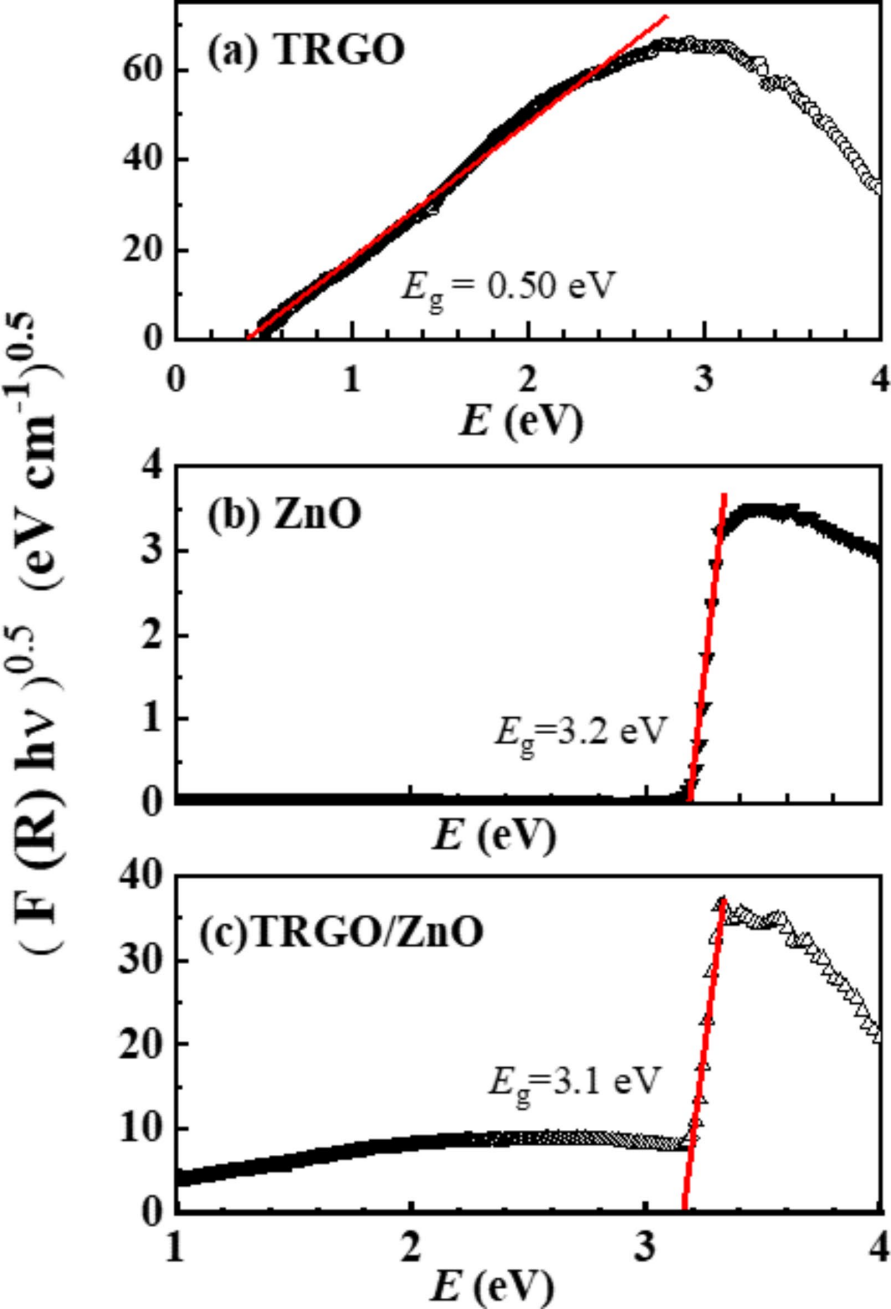
The Kubelka-Munk function versus energy plots for determination of the band gap of (**a**) TRGO, (**b**) ZnO, and (**c**) TRGO/ZnO samples.

Figure 8 (a-c) depicts the energy-dependent behavior of the Kubelka-Munk function, represented, $\:{\left(F\left(R\right)h?\right)}^{1/2}$, for TRGO, ZnO, and TRGO/ZnO samples. Notably, the absorption coefficient remains relatively constant at low energies, up to 3 eV, for both ZnO and TRGO/ZnO nanocomposite. Through analysis of Fig. 8, the band gap energies were determined by identifying the points of intersection with zero values $\:{\left(F\left(R\right)h?\right)}^{1/2}$ on the energy axis. It is observed from Fig. 8 (a) that the TRGO sample exhibits a band gap of 0.50 eV, suggesting the presence of functional groups that differentiate it from graphene’s inherent zero-band gap energy. Figure 8 (b) confirmed the direct band gap energy of ZnO to be 3.2 eV which agrees with the previous study about the optical properties of ZnO nanoparticles^75,76^. From Fig. 8(c) the optical band gap energy of the TRGO/ZnO is direct and equal to 3.1 eV. This indicates that TRGO reduced the band gap energy of ZnO from 3.2 eV to 3.1 eV in the TRGO/ZnO nanocomposite.

### Biological results

#### Antimicrobial activity

**Fig. 9:**
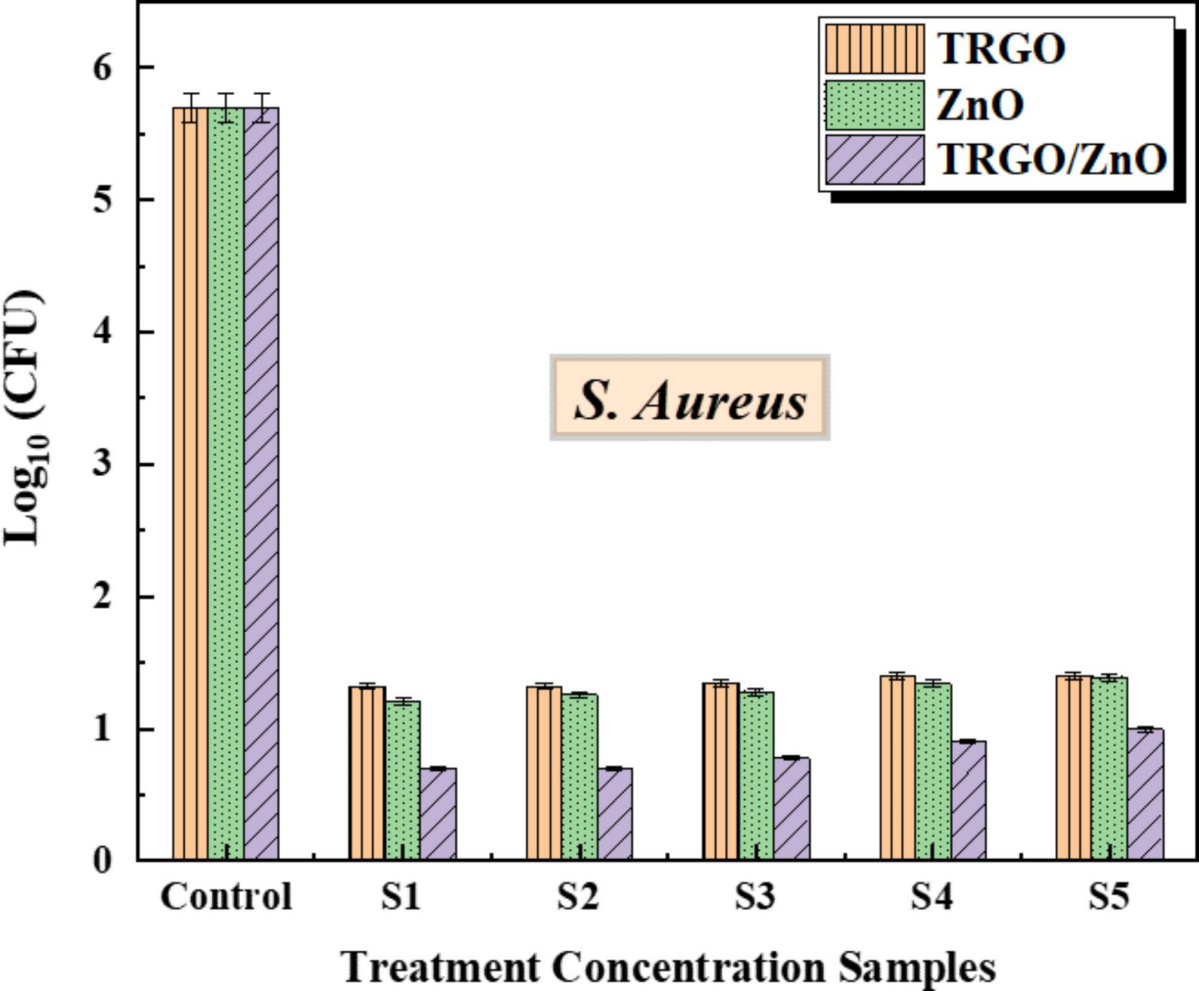
The antibacterial properties of TRGO, ZnO, and TRGO/ZnO samples against *S. aureus*, the untreated control had no treatments. Data from three replications are expressed as mean. The f-ratio value is 54.31126. The p-value is < 0.00001. The result is significant at *p* < .05.

**Fig. 10:**
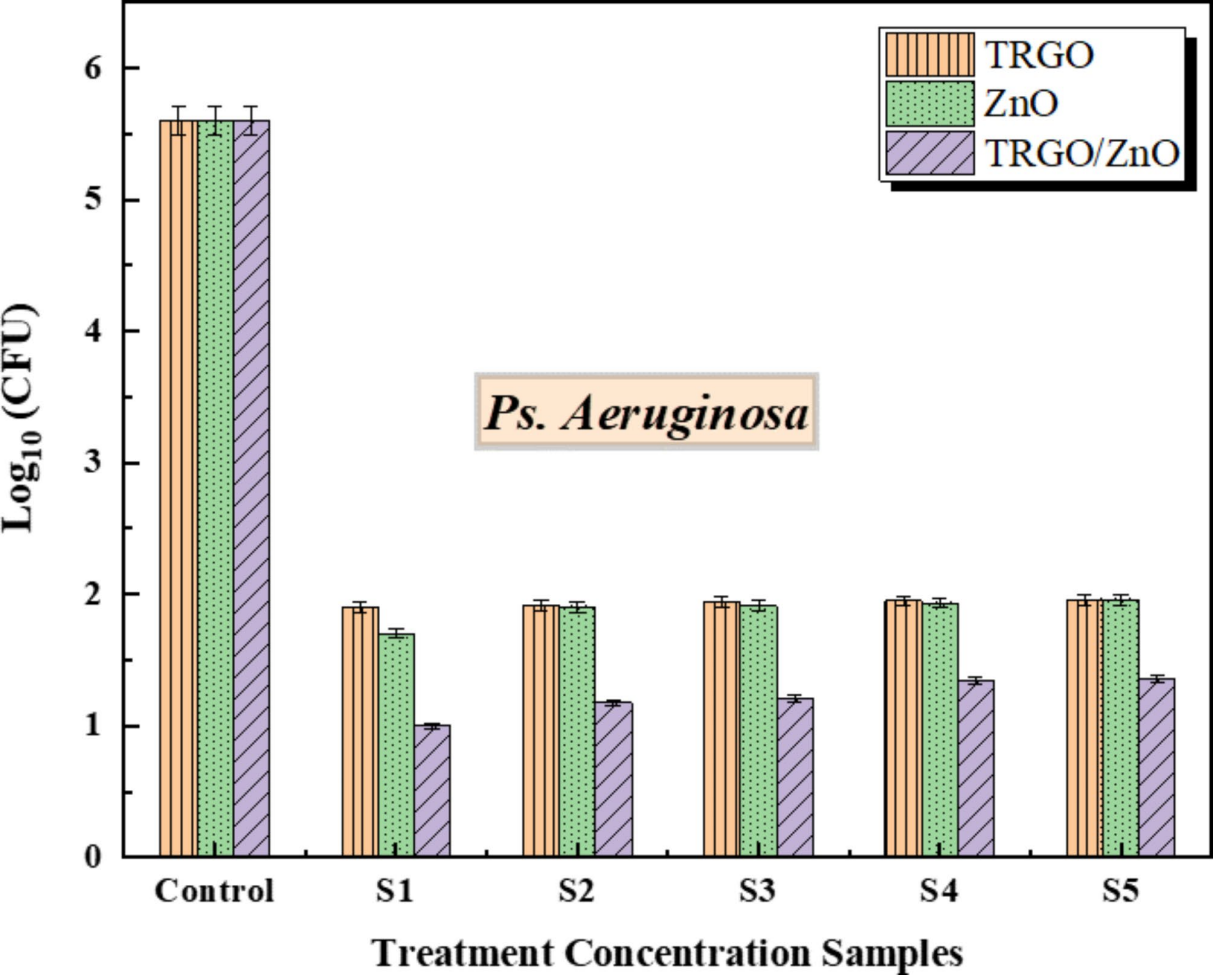
The antibacterial properties of TRGO, ZnO, and TRGO/ZnO samples against *Ps. aeruginosa*, the untreated control had no treatments. Data from three replications are expressed as mean. The f-ratio value is 73.22974. The p-value is < 0.00001. The result is significant at *p* < .05.

**Fig. 11:**
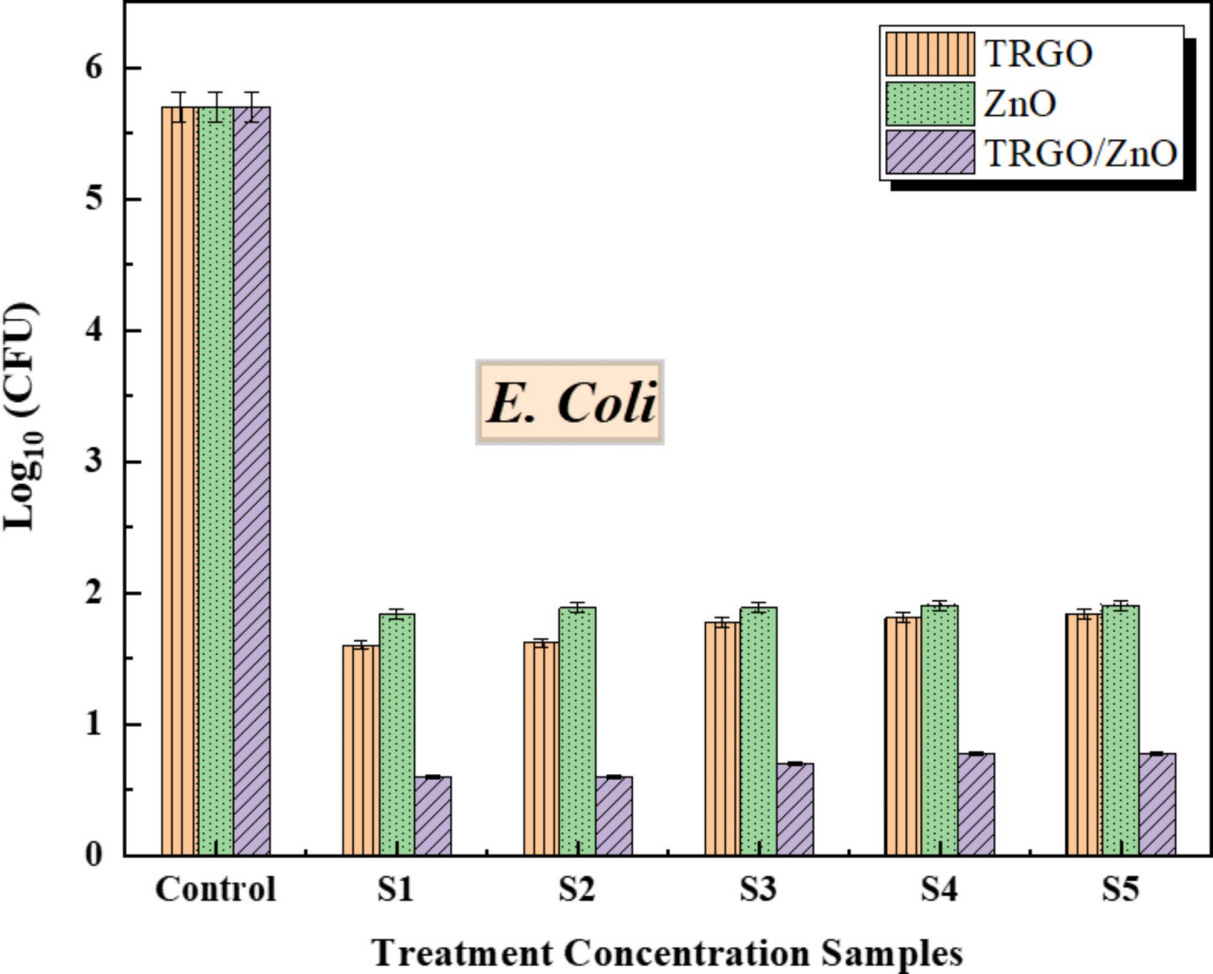
The antibacterial properties of TRGO, ZnO and TRGO/ZnO samples against *E. coli*, the untreated control had no treatments. Data from three replications are expressed as mean. The f-ratio value is 303.02456. The p-value is < 0.00001. The result is significant at *p* < .05.

We applied 0.2 g of the three nanoparticles to cultures of *E. coli*, *Ps. aeruginosa*, and *S. aureus*, followed by serial dilution to achieve final concentrations of 0.01, 0.005, 0.0025, 0.00125, and 0.000625 g/mL for each formulation, designated as S1, S2, S3, S4, and S5 respectively. Bacterial growth was then monitored over 24 h. The results revealed a significant difference in bacterial growth among the three formulations (as shown in Figs. 9 and 10, and 11) at a significance level of *p* < .05. TRGO/ZnO nanoparticles demonstrated superior suppression of bacterial growth, particularly against *S. aureus* and *E. coli*, compared to *Ps. aeruginosa*, especially notable at a concentration of 100 µg/ml.

**Screening antibacterial activity against*S. aureus***.

Showing the control ratio between the thermally reduced graphene oxide TRGO, zinc oxide nanoparticle’s ZnO, and TRGO/ZnO in gram-positive bacteria *S. aureus*. while *Staphylococcus aureus* usually acts as a commensal bacterium, asymptomatically colonizing about 30% of the human population, it can sometimes cause disease. In particular, *S. aureus* is one of the most common causes of bacteremia and infective endocarditis. Additionally, it can cause various skin and soft-tissue infections, particularly when skin or mucosal barriers have been breached. *S. aureus* infections can spread through contact with pus from an infected wound, skin-to-skin contact with an infected person and contact with objects used by an infected person such as towels, sheets, clothing, or athletic equipment.

Figure 9 illustrates the impact of various treatment concentrations of the three prepared nanomaterials on *Staphylococcus aureus* bacteria. The control group exhibited a reduction in bacterial growth compared to the other treatment concentrations. Interestingly, the treatment concentrations of TRGO and ZnO materials remained consistent without significant alterations. However, the different treatment concentrations of the TRGO/ZnO composite exerted varying effects. Higher concentrations, such as S1, demonstrated efficient treatment efficacy, whereas lower concentrations exhibited minimal impact on *S. aureus* bacterial growth.

**Screening antibacterial activity against*Ps. aeruginosa***.

Comparing the control ratio among thermally reduced graphene oxide, zinc oxide nanoparticles, and TRGO/ZnO in gram-negative bacteria *Ps. aeruginosa*, it is noteworthy that this bacterium is frequently encountered in various environmental niches such as soil and water. Among the diverse array of *Pseudomonas* species, *Pseudomonas aeruginosa* is the predominant pathogen causing infections in humans. It is associated with a range of infections, including bloodstream infections, pneumonia, and surgical site infections affecting various anatomical sites.

Figure 10 depicts the impact of the three nanomaterials utilized in this study on the growth of *Ps. aeruginosa* bacteria. A comparison between control samples and different treatment concentrations of those nanomaterials reveals a disruption in bacterial growth induced by the applied treatment concentration of those materials. Interestingly, varying treatment concentrations of TRGO exhibit constant responses to bacterial growth, whereas both ZnO and TRGO/ZnO composite materials demonstrate concentration-dependent effects. Specifically, higher treatment concentrations, such as S1, exhibit the most pronounced disruption of bacterial growth, whereas lower concentrations yield comparatively lesser effects, with a clear concentration-response relationship observed. Furthermore, although both ZnO and composite samples exhibit similar trends, the TRGO/ZnO composite material demonstrates a higher level of disruption compared to ZnO treatment concentrations.

** Screening antibacterial activity against*E. coli***.

Comparing the control ratio among thermal reduced graphene oxide, zinc oxide nanoparticles, and TRGO/ZnO in gram-negative bacteria *Escherichia coli*, which are commonly found in environmental settings, foods, and the intestines of both humans and animals. *E. coli* comprises a broad and diverse group of bacteria. While most *E. coli* strains are benign, certain strains can induce illness. Some variations of *E. coli* are associated with diarrhoea, while others can lead to urinary tract infections, respiratory illnesses such as pneumonia, and various other diseases.

Figure 11 illustrates the control ratio of three nanomaterials in *E. coli*, indicating reductions in bacterial growth across various concentration levels. Specifically, in S1, TRGO/ZnO demonstrates superior control over bacterial growth compared to TRGO and ZnO. For S2, S3, and S4, the TRGO/ZnO composite exhibits the most effective control, followed by TRGO and ZnO.

The current study revealed that the three formulations of TRGO, ZnO, and TRGO/ZnO have promising antimicrobial activity against Gram-positive (*S. aureus*) and Gram-negative (*E. coli*,* Ps. aeruginosa*) bacteria. In Figs. 9, 10, 11. we found that thermal reduced graphene oxide has antibacterial activity. Inorganic metal oxides are commonly employed in biological applications because of their stability and biocompatibility. Less-sized nanoparticles exhibit excellent antibacterial activity and can enter bacterial cells via cell surface holes. A few inorganic nanoparticles, such as CuO, ZnO, TiO_2_, CaO, and Al_2_O_3_, are tested against bacteria. These antibacterial compounds substantially inhibit bacterial growth^77^. Nanomaterials are more effective than traditional antibiotics. Treating infections using nanomaterials will not give rise to resistant bacterial strains^78^. Graphene is a promising antibacterial material due to its nano-size, one atomic thickness and a two-dimensional sheet of sp2 hybridized carbon atoms with a hexagonal honeycomb lattice structure^79^.

#### Biofilm reduction activity of TRGO, ZnO, and TRGO /ZnO nanocomposite

**Fig. 12:**
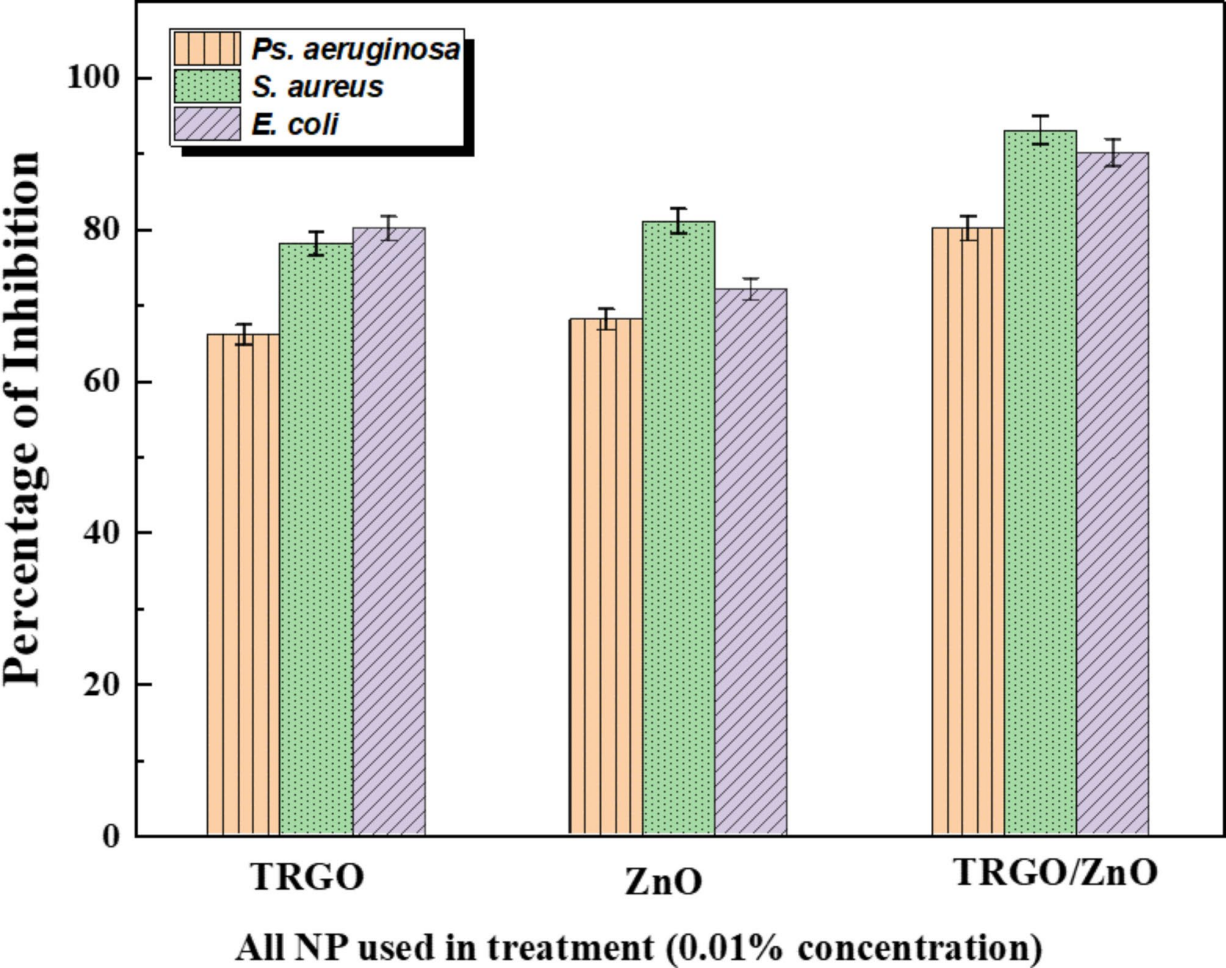
The activity of TRGO, ZnO, and TRGO/ZnO samples against biofilm development utilizing a concentration of 0.01%.

The effectiveness of three nanoparticle formulations against biofilm formation in the bacterial species *S. aureus*, *Ps. aeruginosa*, and *E. coli* was examined as shown in Fig. 12. Each formulation, consisting of TRGO, ZnO, and TRGO/ZnO, was tested at a concentration of 0.01%. Results indicated a significant reduction in biofilm formation for all three bacterial species at this concentration of nanoparticles. Particularly noteworthy was the greater susceptibility of *S. aureus* and *E. coli* compared to *Ps. aeruginosa*. TRGO/ZnO nanoparticles demonstrated the highest anti-biofilm activity among the formulations, achieving a reduction of 93% and 90% against *S. aureus* and *E. coli*, respectively.

The primary cause of non-healing wounds is attributed to biofilms, aggregates of bacteria adhered to wound surfaces. Despite antibiotic therapy, wound infections can persist chronically due to the increasing resistance of biofilms to antibiotics. Our research demonstrated the anti-biofilm activity of the three nanoparticle formulations TRGO, ZnO, and TRGO/ZnO. While TRGO/ZnO exhibited superior bioactivity compared to the other two formulations, all three showed efficacies against biofilms. This activity may be attributed to the nanoparticles encapsulating bacterial cells entirely, potentially altering their morphology or interfering with metabolic activities, thus impeding biofilm development^80^. Additionally, variations in chemical properties and attack mechanisms may influence the association and interactions of the nanocomposites with biofilm-producing bacteria^81,82^.

**Table 1:**
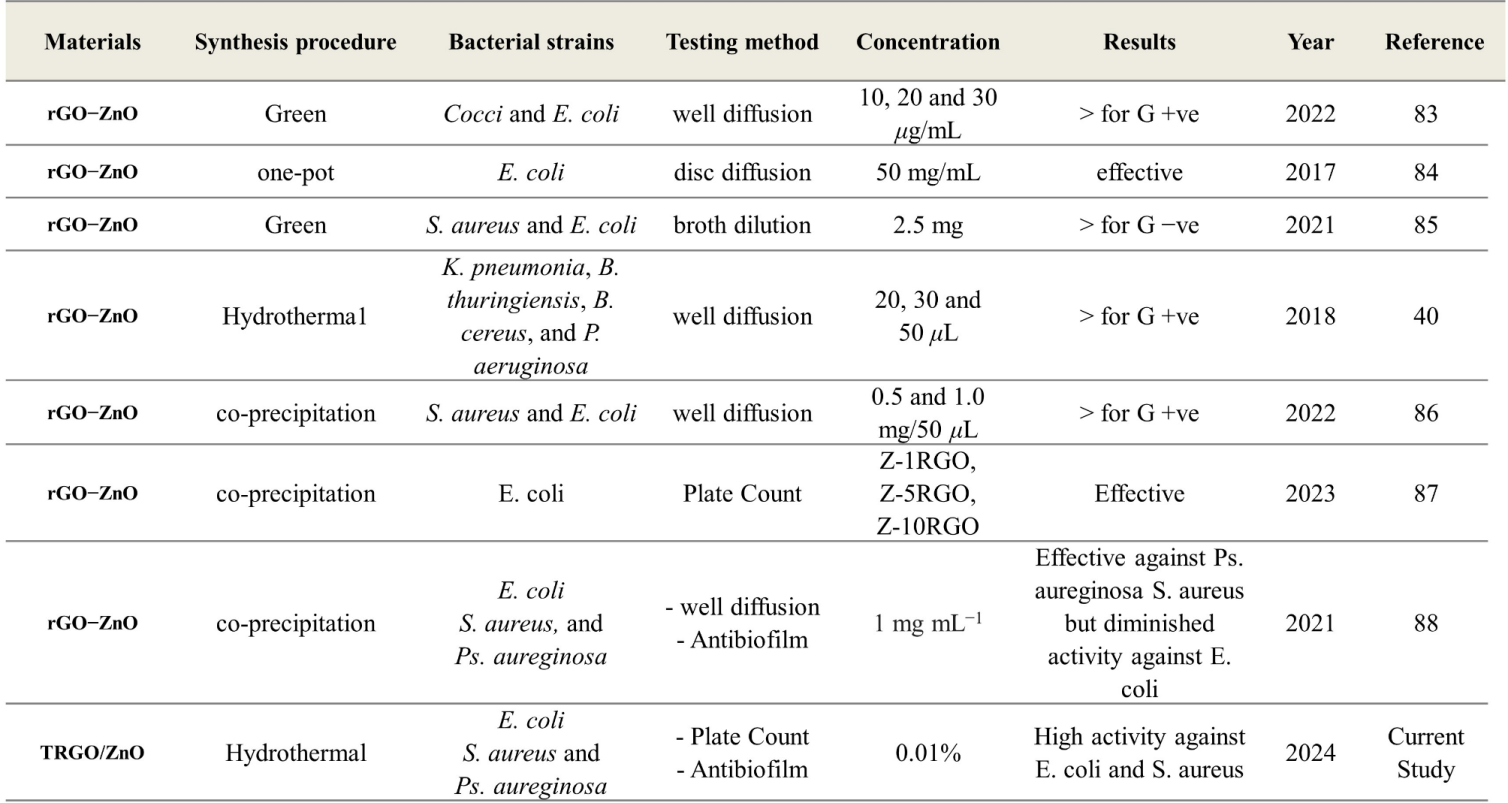
Comparative analysis of the antimicrobial activity of TRGO/ZnO hybrid nanostructure in literature and current research.

Through a comparative analysis of the antimicrobial activity of TRGO/ZnO hybrid nanostructures in existing literature and current research, as shown in Table 1, it is hypothesized that the enhanced antibacterial and anti-biofilm properties of TRGO/ZnO against *E. coli*,* S. aureus*, and *Ps. aeruginosa* are due to the novel manufacturing process of the nanocomposite using the hydrothermal technique. This process results in a lower amount of ZnO particles decorating the reduced graphene sheet compared to other methods. Additionally, the antibacterial and anti-biofilm activity remains high even at concentrations below 0.01%. This research is crucial for future wound-healing applications, particularly highlighting the importance of evaluating the antibiofilm activity of this innovative wound-healing formula.

### Reactive oxygen species (ROS) assay

**Fig. 13:**
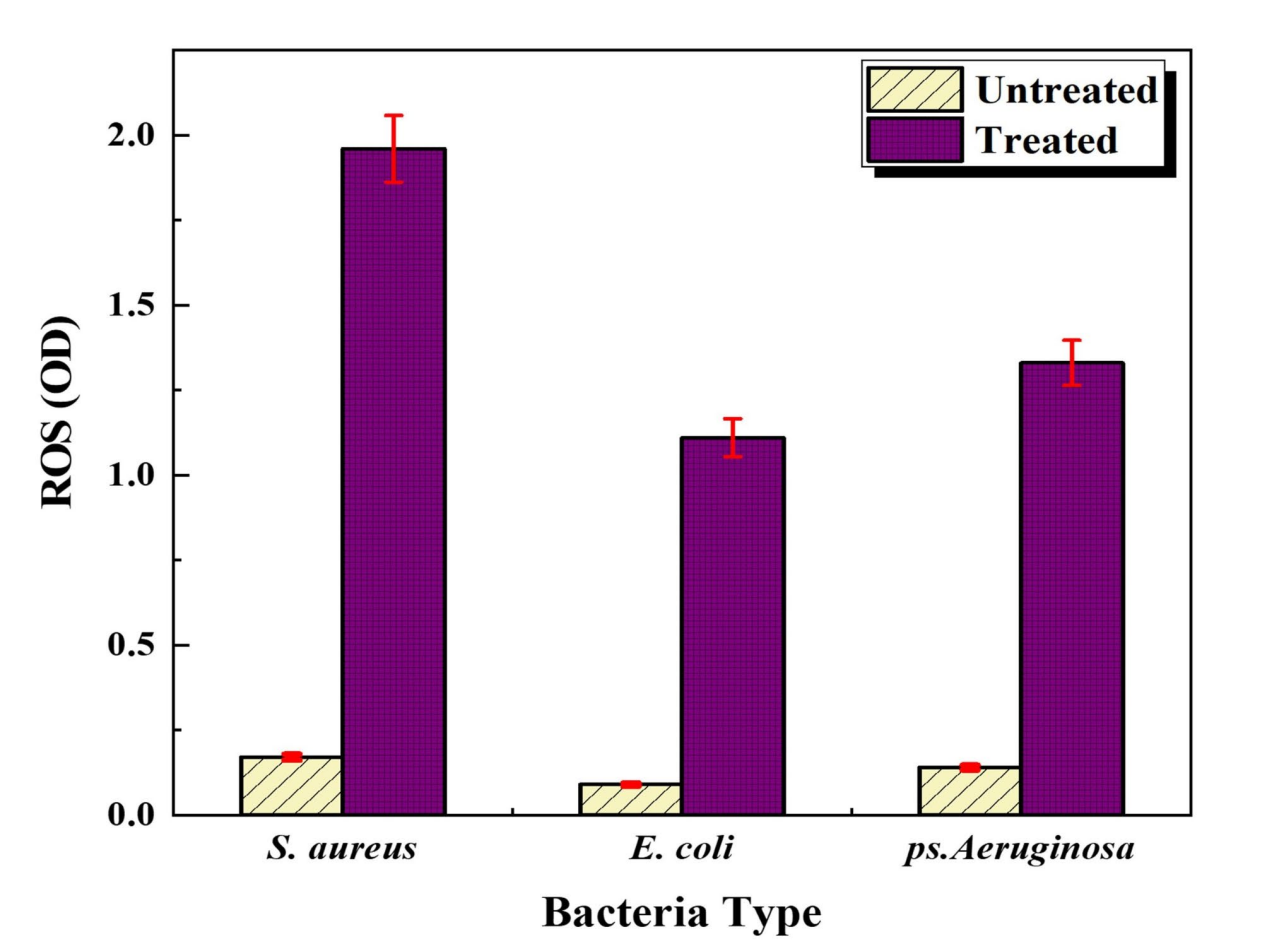
ROS analysis of TRGO/ZnO nanocomposites in *S. aureus*,* E. coli*, and *Ps. aeruginosa*.

Reactive oxygen species (ROS) analysis is the most effective method for determining the toxicity of nanocomposites. The intracellular ROS generation was evaluated with the oxidant-detecting fluorescent dye DCFH-DA (2′,7′-Dichlorodihydrofluorescein diacetate). ROS is typically produced in the mitochondria of cells. It’s a byproduct of cellular oxidative metabolism. ROS include hydroxyl radicals, hydrogen peroxide, superoxide anion radicals, and singlet oxygen^89^. An increase in ROS production induces oxidative stress, which causes cell disruption and damage. It will alter the mechanism of protein radical formation, lipid peroxidation, DNA strand breaking, signal transduction regulation, and cell death. ROS molecules penetrate the cell membrane, aggregate within the cells, and trigger apoptosis. Figure 13 depicts the increase in ROS formation with the TRGO/ZnO nanocomposite as compared to the control in the three microorganisms *S. aureus*,* E. coli*, and *Ps. aeruginosa*.

. Graphene Oxide was proven to cause oxidative stress (OS) in bacterial cells due to its structure and physiochemical characteristics, while, TRGO/ZnO nanocomposite increases ROS generation in all bacterial strains as compared to TRGO due to the activation of the NADPH oxidase enzyme when ZnO comes into touch with a bacterial cell, it disrupts its integrity by releasing Zn^2+^ ions^90,91^. As a result, TRGO/ZnO nanocomposites suppress bacteria by producing ROS.

#### Evaluation of cytotoxicity and cell viability of TRGO/ZnO nanocomposite (MTT assay)

**Fig. 14:**
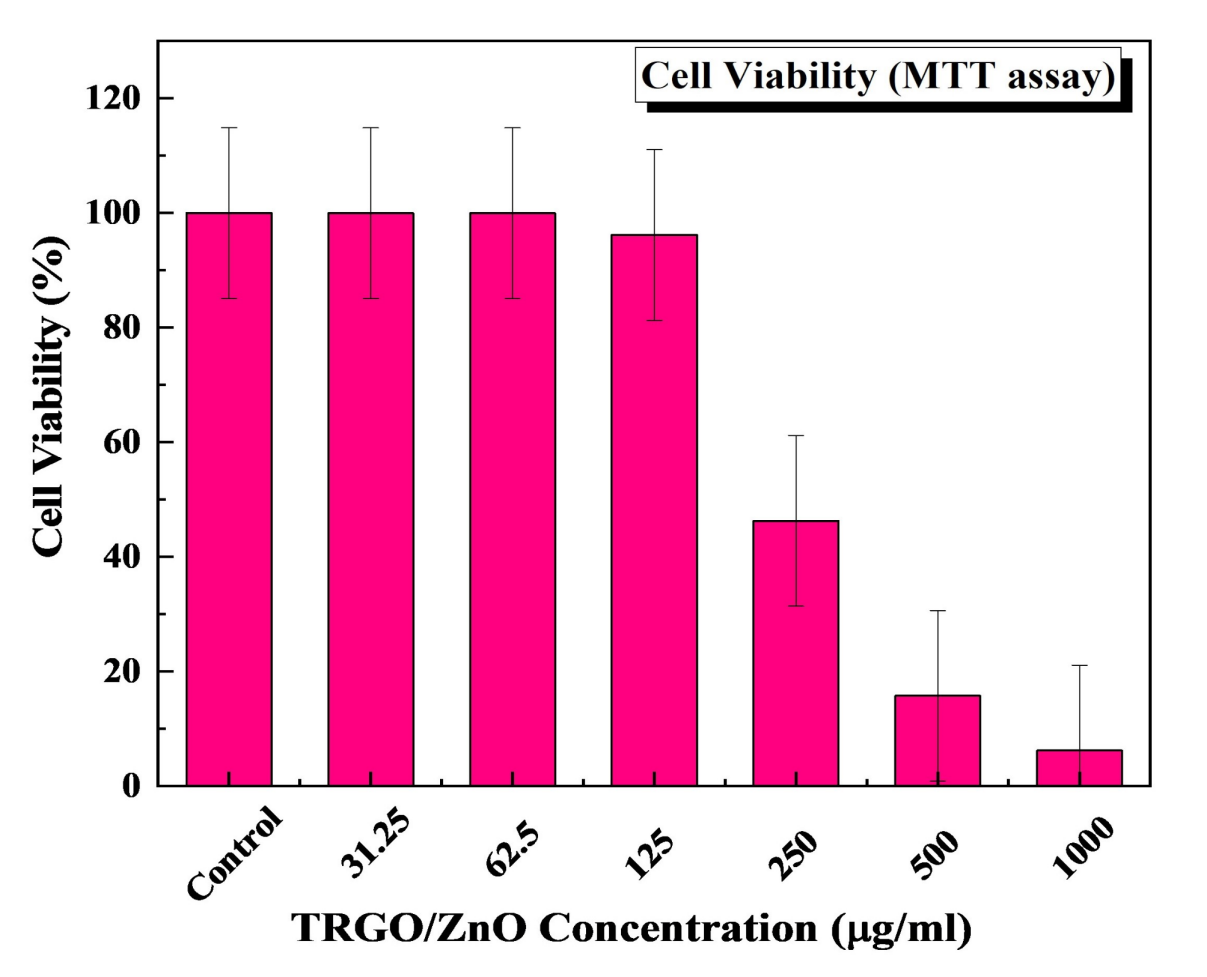
Evaluation of the cytotoxicity of TRGO/ZnO nanocomposite against Normal Human Epidermal Keratinocytes (NHEK) cells.

The MTT assay was used to assess the viability of NHEK cells exposed to TRGO/ZnO nanocomposite in order to conduct cytotoxicity assays 24 h after the nanocomposite treatment. As shown in Fig. 14, 24 h after the incubation, the cell viability of groups with TRGO/ZnO concentration less than 125 µg/mL was higher than 96.16% of the control group. This suggested that TRGO/ZnO nanocomposite is biocompatible and has minimal cytotoxicity. To date, investigations on the cytotoxicity of TRGO have been conflicting. While some research indicates that TRGO has no harmful effects on cellular functioning, other studies claim that this nanomaterial can cause harm to cells. Research has demonstrated that by enhancing mammalian cell adhesion and proliferation, TRGO can greatly stimulate cell growth^92^. According to other research, TRGO has good biocompatibility and can effectively promote cell adhesion and proliferation. It is possible to decipher TRGO’s beneficial interactions with cells from its chemical structure. The abundant oxygen-containing functional groups have been proposed to be in charge of providing sufficient support for cell adhesion and proliferation^93^. Additionally, it has been discovered that TRGO efficiently supplies soluble components and essential signals for cell growth and adhesion^94^. Furthermore, the physical features of TRGO, such as shape, particle size, number of layers, and surface functionalization, influence its effect on cells. For example, TRGO at a dose less than 20 µg/mL did not exhibit toxicity to human fibroblast cells, but a dose more than 50 µg/mL exhibits cytotoxicity such as lowering cell adhesion, triggering cell apoptosis, and entering into lysosomes, mitochondrion, endoplasm, and the cell nucleus^95^. On the contrary to the present study, a concentration of 125 µg/mL (which is greater than the effective antimicrobial dose) showes very low toxicity and high biocompatabilty with keratinocyte cells which may be due to the new method used in nanocomposite preparation.

## Conclusions

The nanocomposites, featuring a combination of thermally reduced graphene oxide and zinc oxide (TRGO/ZnO), were successfully synthesized utilizing a hydrothermal method. A comprehensive investigation of their physicochemical characteristics was conducted using XRD, FT-IR spectroscopy, FE-SEM, HR-TEM, Raman spectroscopy, and UV-vis spectroscopy. Distinctly captured in FE-SEM and HR-TEM images were spherical ZnO nanoparticles embellishing the surfaces of graphene sheets. FT-IR analysis confirmed the incomplete reduction of oxygen in graphene oxide, underscoring the presence of oxygen functional groups. Furthermore, the Raman spectroscopy confirms the incorporation of ZnO onto the TRGO surface, with this decoration leading to a reduction in defects on the TRGO. This suggests that the interaction between ZnO and TRGO has a stabilizing effect on the graphene structure. The exploration of optical properties unveiled that TRGO induced a reduction in the band gap energy of ZnO from 3.20 eV to 3.1 eV within the TRGO/ZnO nanocomposites.

Furthermore, the potential of TRGO/ZnO nanocomposites as bio-active materials against bacteria responsible for wound infections, including *Staphylococcus aureus*, *Pseudomonas aeruginosa*, and *Escherichia coli*, was evaluated for medical applications. Impressively, the tested samples exhibited the capability to disrupt bacterial biofilm formation. Evaluation of both antibiofilm and antimicrobial activity is crucial when assessing bioactivity in the context of wound healing. This study, one of the relatively few that emphasize nanocomposite antibiofilm activity, demonstrated that the novel preparation technique resulted in high levels of both antimicrobial and antibiofilm activity at a concentration lower than all previously published research, particularly against *E. coli* and *S. aureus* with proven high biocompatibility. These compelling findings strongly suggest that TRGO/ZnO nanocomposite samples hold substantial promise as highly effective safe bio-active nanomaterials, offering a potent solution for combating pathogenic microorganisms commonly encountered in wound-related scenarios.

## Data Availability

The data that support the findings of this study are available from the corresponding author upon reasonable request.

## References

[1] Mohd Ariffin, N. H. & Hasham, R. Assessment of non-invasive techniques and herbal-based products on dermatological physiology and intercellular lipid properties. *Heliyon*. **6** (5), e03955 (2020). 32478187 10.1016/j.heliyon.2020.e03955 PMC7251381

[2] Scalise, A. et al. Microenvironment and microbiology of skin wounds: the role of bacterial biofilms and related factors. in Seminars in Vascular Surgery. Elsevier. (2015). 10.1053/j.semvascsurg.2016.01.003 27113281

[3] Boateng, J. & Catanzano, O. Advanced Therapeutic dressings for Effective Wound Healing—A Review. *J. Pharm. Sci.* **104** (11), 3653–3680 (2015). 26308473 10.1002/jps.24610

[4] Robson, M. C. WOUND INFECTION: a failure of Wound Healing caused by an imbalance of Bacteria. *Surg. Clin. North Am.* **77** (3), 637–650 (1997). 9194884 10.1016/s0039-6109(05)70572-7

[5] Jahromi, M. A. M. et al. Nanomedicine and advanced technologies for burns: preventing infection and facilitating wound healing. *Adv. Drug Deliv. Rev.* **123**, 33–64 (2018). 28782570 10.1016/j.addr.2017.08.001 PMC5742034

[6] Zhao, Y. et al. *Advanced Bioactive Nanomaterials for Biomedical Applications. In Exploration* (Wiley Online Library, 2021). 10.1002/EXP.20210089 PMC10191050 37323697

[7] Barkalina, N. et al. Nanotechnology in reproductive medicine: emerging applications of nanomaterials. *Nanomed. Nanotechnol. Biol. Med.* **10** (5), e921–e938 (2014). 10.1016/j.nano.2014.01.001 24444494

[8] Gold, K. et al. Antimicrobial activity of metal and metal-oxide based nanoparticles. *Adv. Ther.* **1** (3), 1700033 (2018).

[9] Ye, L. et al. Noble metal-based nanomaterials as antibacterial agents. *J. Alloys Compd.* **904**, 164091 (2022).

[10] El-Nahrawy, A. M. et al. Influences of Ag-NPs doping chitosan/calcium silicate nanocomposites for optical and antibacterial activity. *Int. J. Biol. Macromol.* **93**, 267–275 (2016). 27543348 10.1016/j.ijbiomac.2016.08.045

[11] Ren, R. et al. Recent advances in the development of lipid-, metal-, carbon-, and polymer-based nanomaterials for antibacterial applications. *Nanomaterials*. **12** (21), 3855 (2022). 36364631 10.3390/nano12213855 PMC9658259

[12] Fritea, L. et al. Metal nanoparticles and carbon-based nanomaterials for improved performances of electrochemical (Bio) sensors with biomedical applications. *Materials*. **14** (21), 6319 (2021). 34771844 10.3390/ma14216319 PMC8585379

[13] Rozbu, M. R., Kabir, A. & Selvakumar, P. M. Functionalized Bio-carbon Nanomaterials for Environmental Utilizations *Environ. Appli. Carbon Nanomater. Based Devices*. 347–374 (2021).

[14] Augustine, R. et al. Electrospun Chitosan membranes containing bioactive and therapeutic agents for enhanced wound healing. *Int. J. Biol. Macromol.* **156**, 153–170 (2020). 32229203 10.1016/j.ijbiomac.2020.03.207

[15] Zhang, X. M. et al. *Multifunctional polydopamine/hemin-cyclodextrin reinforced chitosan nanocomposite hydrogel: a synergistic platform for wound healing*. *Int. J. Biol. Macromol.* **260** 129553. (2024). 10.1016/j.ijbiomac.2024.129553 38246439

[16] Pino, P. et al. Antimicrobial nano-zinc oxide biocomposites for wound healing applications: a review. *Pharmaceutics*. **15** (3), 970 (2023). 36986831 10.3390/pharmaceutics15030970 PMC10053511

[17] Pulit-Prociak, J. et al. Analysis of the physicochemical properties of antimicrobial compositions with zinc oxide nanoparticles. *Sci. Technol. Adv. Mater.* **20** (1), 1150–1163 (2019). 32082437 10.1080/14686996.2019.1697617 PMC7006636

[18] Laurenti, M. & Cauda, V. ZnO nanostructures for tissue engineering applications. *Nanomaterials*. **7** (11), 374 (2017). 29113133 10.3390/nano7110374 PMC5707591

[19] Wiesmann, N. et al. Zinc oxide nanoparticles exhibit favorable properties to promote tissue integration of biomaterials. *Biomedicines*. **9** (10), 1462 (2021). 34680579 10.3390/biomedicines9101462 PMC8533365

[20] Nethi, S. K. et al. Recent advances in inorganic nanomaterials for wound-healing applications. *Biomaterials Sci.* **7** (7), 2652–2674 (2019). 10.1039/c9bm00423h 31094374

[21] Ferrone, E. et al. ZnO nanostructures and electrospun ZnO–polymeric hybrid nanomaterials in biomedical, health, and sustainability applications. *Nanomaterials*. **9** (10), 1449 (2019). 31614707 10.3390/nano9101449 PMC6835458

[22] Liao, C., Li, Y. & Tjong, S. C. Graphene nanomaterials: synthesis, biocompatibility, and cytotoxicity. *Int. J. Mol. Sci.* **19** (11), 3564 (2018). 30424535 10.3390/ijms19113564 PMC6274822

[23] Gurunathan, S. & Kim, J. H. *Synthesis, toxicity, biocompatibility, and biomedical applications of graphene and graphene-related materials*. *Int. J. Nanomed.* **11**, 1927–1945. (2016). 10.2147/IJN.S105264 PMC4863686 27226713

[24] Pulingam, T. et al. Mechanistic actions and contributing factors affecting the antibacterial property and cytotoxicity of graphene oxide. *Chemosphere*. **281**, 130739 (2021). 34004516 10.1016/j.chemosphere.2021.130739

[25] Seifi, T. & Kamali, A. R. Anti-pathogenic activity of graphene nanomaterials: a review. *Colloids Surf., B*. **199**, 111509 (2021). 10.1016/j.colsurfb.2020.111509 33340933

[26] Kumar, P. et al. Antibacterial properties of graphene-based nanomaterials. *Nanomaterials*. **9** (5), 737 (2019). 31086043 10.3390/nano9050737 PMC6567318

[27] Ji, H., Sun, H. & Qu, X. Antibacterial applications of graphene-based nanomaterials: recent achievements and challenges. *Adv. Drug Deliv. Rev.* **105**, 176–189 (2016). 27129441 10.1016/j.addr.2016.04.009

[28] Maio, A. et al. An overview of functionalized graphene nanomaterials for advanced applications. *Nanomaterials*. **11** (7), 1717 (2021). 34209928 10.3390/nano11071717 PMC8308136

[29] Azizi-Lalabadi, M. et al. Carbon nanomaterials against pathogens; the antimicrobial activity of carbon nanotubes, graphene/graphene oxide, fullerenes, and their nanocomposites. *Adv. Colloid Interface Sci.* **284**, 102250 (2020). 32966964 10.1016/j.cis.2020.102250

[30] Xin, Q. et al. Antibacterial carbon-based nanomaterials. *Adv. Mater.* **31** (45), 1804838 (2019). 10.1002/adma.201804838 30379355

[31] Díez-Pascual, A. M. Carbon-based nanomaterials*MDPI*. p: 7726. (2021). 10.3390/ijms22147726 PMC8307333 34299346

[32] Ibraheem, D. R. et al. Ciprofloxacin-loaded silver nanoparticles as potent nano-antibiotics against resistant pathogenic bacteria. *Nanomaterials*. **12** (16), 2808 (2022). 36014673 10.3390/nano12162808 PMC9415342

[33] Thuy, D. B. et al. Colonization with Staphylococcus aureus and Klebsiella pneumoniae causes infections in a Vietnamese intensive care unit. *Microb. Genomics*. **7** (2), 000514 (2021). 10.1099/mgen.0.000514 PMC8208697 33502303

[34] Saleemi, M. A. et al. An overview of antimicrobial properties of carbon nanotubes-based nanocomposites. *Adv. Pharm. Bull.* **12** (3), 449 (2022). 35935059 10.34172/apb.2022.049 PMC9348533

[35] Malet, D. *Biotechnology and International Security* (Rowman & Littlefield, 2016).

[36] Warner, C. M. *Rapid, large-scale production of full-length, human-like monoclonal antibodies.* (2012).

[37] Raslan, A. et al. Graphene oxide and reduced graphene oxide-based scaffolds in regenerative medicine. *Int. J. Pharm.* **580**, 119226 (2020). 32179151 10.1016/j.ijpharm.2020.119226

[38] Angulo-Pineda, C. et al. Electroactive 3D printed scaffolds based on percolated composites of polycaprolactone with thermally reduced graphene oxide for antibacterial and tissue engineering applications. *Nanomaterials*. **10** (3), 428 (2020). 32121237 10.3390/nano10030428 PMC7152842

[39] Alemi, F. et al. Graphene oxide and reduced graphene oxide: efficient cargo platforms for cancer theranostics. *J. Drug Deliv. Sci. Technol.* **60**, 101974 (2020).

[40] Rajeswari, R. & Prabu, H. G. Synthesis characterization, antimicrobial, antioxidant, and cytotoxic activities of ZnO nanorods on reduced graphene oxide. *J. Inorg. Organomet. Polym Mater.* **28**, 679–693 (2018).

[41] Sengupta, I. et al. Bactericidal effect of graphene oxide and reduced graphene oxide: influence of shape of bacteria. *Colloid Interface Sci. Commun.* **28**, 60–68 (2019).

[42] El-Basaty, A. B., Moustafa, E., Fouda, A. N. & El-Moneim, A. A. 3D hierarchical graphene/CNT with interfacial polymerized polyaniline nano-fibers. *Spectrochim. Acta Part A Mol. Biomol. Spectrosc.* **226**, 117629 (2020). 10.1016/j.saa.2019.117629 31606670

[43] Riyadh, M. et al. Synthesis of Zinc Oxide nanoparticles via Sol – Gel Route and their characterization. *Nanosci. Nanatechnol.* **5** (1), 1–6 (2015).

[44] Avinash, R. et al. Zinc Oxide/Graphene oxide nanocomposites: synthesis, characterization and their Optical Properties. *ES Mater. Manuf.* **16**, 19–29 (2022).

[45] Quirk, T. J. & Quirk, T. J. One-way analysis of variance (ANOVA).*Excel 2007 Educ. Psychol. Stat. Guide Solving Pract. Probl.*. pp. 163–179. (2012).

[46] Kim, T. K. Understanding one-way ANOVA using conceptual figures. *Korean J. Anesthesiol.* **70** (1), 22 (2017). 10.4097/kjae.2017.70.1.22 PMC5296382 28184262

[47] Akande, R. T. et al. Anthelmintic and antimycobacterial activity of fractions and compounds isolated from Cissampelos mucronata. *SSRN*. p. 22. (2022). 10.1016/j.jep.2022.115130 35292375

[48] Baskar, K., Anusuya, T. & Venkatasubbu, G. D. Mechanistic investigation on microbial toxicity of nano hydroxyapatite on implant associated pathogens. *Mater. Sci. Engineering: C*. **73**, 8–14 (2017). 10.1016/j.msec.2016.12.060 28183675

[49] Wang, M. et al. *A comparative study of toxicity of TiO2, ZnO, and Ag nanoparticles to human aortic smooth-muscle cells*. *Int. J. Nanomed.* **13** pp. 8037–8049. (2018). 10.2147/IJN.S188175 PMC6267729 30568444

[50] Said, A. A. E. et al. Abdelrahman M. HelmyNiosomes as promising approach for enhancing the cytotoxicity of Hemimycale sp. total crude extract supported with in-silico studies. *Sci. Rep.* **14**, 2546. (2024). 10.1038/s41598-024-52918-3 PMC10827731 38291122

[51] Ali, A. I., Salim, S. A. & Kamoun, E. A. Novel glass materials-based (PVA/PVP/Al2O3/SiO2) hybrid composite hydrogel membranes for industrial applications: synthesis, characterization, and physical properties. *J. Mater. Sci.: Mater. Electron.* **33** (13), 10572–10584 (2022).

[52] Huh, S. H. Thermal reduction of graphene oxide. *Phys. Appl. Graphene Exp.*. **19**, 73–90 (2011).

[53] Gómez-Navarro, C. et al. Atomic structure of reduced graphene oxide. *Nano Lett.* **10** (4), 1144–1148 (2010). 20199057 10.1021/nl9031617

[54] Talam, S., Karumuri, S. R. & Gunnam, N. *Synthesis, characterization, and spectroscopic properties of ZnO nanoparticles.* International Scholarly Research Notices, 2012. (2012).

[55] Janaki, A. C., Sailatha, E. & Gunasekaran, S. Synthesis, characteristics and antimicrobial activity of ZnO nanoparticles. *Spectrochim. Acta Part A Mol. Biomol. Spectrosc.* **144**, 17–22 (2015). 10.1016/j.saa.2015.02.041 25748589

[56] Tian, Z. R. et al. Complex and oriented ZnO nanostructures. *Nat. Mater.* **2** (12), 821–826 (2003). 14634640 10.1038/nmat1014

[57] Sharma, D. K. et al. A review on ZnO: Fundamental properties and applications.* Mater.Today Proc.* **49**, 3028–3035. (2022).

[58] Hosono, E. et al. Non-basic solution routes to prepare ZnO nanoparticles. *J. Solgel Sci. Technol.* **29**, 71–79 (2004).

[59] Niyitanga, T. & Kim, H. Reduced graphene oxide supported zinc/cobalt oxide nanoparticles as highly efficient electrocatalyst for oxygen evolution reaction. *Inorg. Chim. Acta*. **539**, 121008 (2022).

[60] Youssry, S. M. et al. Thermal-assisted synthesis of reduced graphene oxide-embedded Ni nanoparticles as high-performance electrode material for supercapacitor. *Electrochim. Acta*. **463**, 142814 (2023).

[61] Huang, S. et al. ZnO nanosheet balls anchored onto graphene foam for electrochemical determination of dopamine in the presence of uric acid. *Sens. Actuators B*. **277**, 381–387 (2018).

[62] Amiri, A. et al. A review on liquid-phase exfoliation for scalable production of pure graphene, wrinkled, crumpled and functionalized graphene and challenges. *FlatChem*. **8**, 40–71 (2018).

[63] Samuel, E. et al. Electrosprayed graphene decorated with ZnO nanoparticles for supercapacitors. *J. Alloys Compd.* **741**, 781–791 (2018).

[64] Narayanam, P. K. Efficient tunability of size and optical properties of reduced graphene oxide-ZnO composite nanocrystallites on solid substrates. *Colloids Surf. A Pysicochem. Eng. Asp.*. **665**, 131229 (2023).

[65] Ganguly, A. et al. Probing the thermal deoxygenation of graphene oxide using high-resolution in situ x-ray-based spectroscopies. *J. Phys. Chem. C*. **115** (34), 17009–17019 (2011).

[66] Diop, C. I. K. et al. Designing bilayered composite films by direct agar/chitosan and citric acid-crosslinked PVA/agar layer-by-layer casting for packaging applications. *Food Hydrocoll.* **144**, 108987 (2023).

[67] Atherton, K., Newbold, G. & Hockey, J. Infra-red spectroscopic studies of zinc oxide surfaces. *Discuss. Faraday Soc.* **52**, 33–43 (1971).

[68] Ashkenov, N. et al. Infrared dielectric functions and phonon modes of high-quality ZnO films. *J. Appl. Phys.* **93** (1), 126–133 (2003).

[69] Noei, H. et al. The identification of hydroxyl groups on ZnO nanoparticles by infrared spectroscopy. *Phys. Chem. Chem. Phys.* **10** (47), 7092–7097 (2008). 19039343 10.1039/b811029h

[70] Bellamy, L. *The infra-red Spectra of Complex Molecules* (Springer Science & Business Media, 2013).

[71] Eley, D., Rochester, C. & Scurrell, M. Polymerization of ethylene on chromium oxide catalysts. Part 4.—Infra-red study of the adsorption of nitric oxide and ammonia on active catalyst. *J. Chem. Soc. Faraday Trans. 1: Phys. Chem. Condens. Phases*. **69**, 660–671 (1973).

[72] Scaltriti, F. et al. Optical polarization properties of GRO J1655-40.*Astron. Astrophys.* **325**, L29–L31. (1997).

[73] Soosen Samuel, M., Bose, L. & George, K. Optical properties of ZnO nanoparticles. *Acad. Rev.* **16**, 57–65 (2009).

[74] Ali, A. I., Abdel, A. & Moez Study of non-linear spectroscopic optical properties, electrical susceptibility and semiconducting dependence on substrate temperature for ZnO thin films prepared by radio frequency technique.*Egypt. J. Chem.* **62**, 137–147. (2019).

[75] Rusdi, R. et al. Preparation and band gap energies of ZnO nanotubes, nanorods and spherical nanostructures. *Powder Technol.* **210** (1), 18–22 (2011).

[76] Musa, I. & QAMHIEH, N. Study of optical energy gap and quantum confinment effects in Zinc Oxide nanoparticles and nanorods. *Dig. J. Nanomater. Biostruct.* **14**, 119-125. (2019).

[77] Tahir, K. et al. Visible light photo catalytic inactivation of bacteria and photo degradation of methylene blue with Ag/TiO2 nanocomposite prepared by a novel method. *J. Photochem. Photobiol., B*. **162**, 189–198 (2016). 27376463 10.1016/j.jphotobiol.2016.06.039

[78] Bhaisare, M. L. et al. MALDI MS analysis, disk diffusion and optical density measurements for the antimicrobial effect of zinc oxide nanorods integrated in graphene oxide nanostructures. *Biomaterials Sci.* **4** (1), 183–194 (2016). 10.1039/c5bm00342c 26575840

[79] Jayanti, D. N., Nugraheni, A. Y. & Baqiya, M. A. *Photoluminescence of reduced graphene oxide prepared from old coconut shell with carbonization process at varying temperatures*. in *IOP conference series: materials science and engineering*. IOP Publishing. (2017).

[80] Selim, M. S. et al. *Facile design of reduced graphene oxide decorated with Cu2O nanocube composite as antibiofilm active material*. *Mater. Chem. Phys.* **239**, 122300 (2020).

[81] El-Batal, A. I. et al. *Nystatin mediated bismuth oxide nano-drug synthesis using gamma rays for increasing the antimicrobial and antibiofilm activities against some pathogenic bacteria and Candida species*. *RSC Adv.* **10**, 9274–9289. (2020). 10.1039/c9ra10765g PMC9050052 35497243

[82] Mahamuni, P. P. et al. Synthesis and characterization of zinc oxide nanoparticles by using polyol chemistry for their antimicrobial and antibiofilm activity. *Biochem. Biophys. Rep.* **17**, 71–80 (2019). 30582010 10.1016/j.bbrep.2018.11.007 PMC6295600

[83] Malik, A. R. et al. Green synthesis of RGO-ZnO mediated Ocimum basilicum leaves extract nanocomposite for antioxidant, antibacterial, antidiabetic and photocatalytic activity. *J. Saudi Chem. Soc.* **26** (2), 101438 (2022).

[84] Rajaura, R. S. et al. Synthesis, characterization and enhanced antimicrobial activity of reduced graphene oxide–zinc oxide nanocomposite. *Mater. Res. Express*. **4** (2), 025401 (2017).

[85] Dhandapani, P. et al. Biological mediated synthesis of RGO-ZnO composites with enhanced photocatalytic and antibacterial activity. *J. Hazard. Mater.* **409**, 124661 (2021). 33288337 10.1016/j.jhazmat.2020.124661

[86] Usman, O. et al. Enhanced bactericidal action of rGO–ZnO hybrids prepared by the One-Pot Co-precipitation Approach. *ACS Omega*. **7** (30), 26715–26722 (2022). 35936465 10.1021/acsomega.2c03049 PMC9352235

[87] Ahmadi, R. et al. A comparative study: green synthesis and evaluation of ZnO-GO and ZnO-RGO nanocomposites for antibacterial applications. *Mater. Sci. Engineering: B*. **294**, 116555 (2023).

[88] Elbasuney, S. et al. Promising antimicrobial and antibiofilm activities of reduced graphene oxide-metal oxide (RGO-NiO, RGO-AgO, and RGO-ZnO) nanocomposites. *RSC Adv.* **11** (42), 25961–25975 (2021). 35479482 10.1039/d1ra04542c PMC9037130

[89] Raghupathi, K. R., Koodali, R. T. & Manna, A. C. Size-dependent bacterial growth inhibition and mechanism of antibacterial activity of zinc oxide nanoparticles. *Langmuir*. **27** (7), 4020–4028 (2011). 21401066 10.1021/la104825u

[90] Chen, D. et al. Graphene-wrapped ZnO nanospheres as a photocatalyst for high performance photocatalysis. *Thin Solid Films*. **574**, 1–9 (2015).

[91] Prema, D. et al. Mechanism of inhibition of graphene oxide/zinc oxide nanocomposite against wound infection causing pathogens. *Appl. Nanosci.* **10**, 827–849 (2020).

[92] Ruiz, O. N. et al. Graphene oxide: a nonspecific enhancer of cellular growth. *ACS nano*. **5** (10), 8100–8107 (2011). 21932790 10.1021/nn202699t

[93] Chen, G. Y. et al. A graphene-based platform for induced pluripotent stem cells culture and differentiation. *Biomaterials*. **33** (2), 418–427 (2012). 22014460 10.1016/j.biomaterials.2011.09.071

[94] Yoon, H. H. et al. Dual roles of graphene oxide in chondrogenic differentiation of adult stem cells: cell-adhesion substrate and growth factor‐delivery carrier. *Adv. Funct. Mater.* **24** (41), 6455–6464 (2014).

[95] Wang, Y. et al. High-quality reduced graphene oxide-nanocrystalline platinum hybrid materials prepared by simultaneous co-reduction of graphene oxide and chloroplatinic acid. *Nanoscale Res. Lett.* **6**, 1–8 (2011). 10.1186/1556-276X-6-241 PMC3211302 21711745

